# Gut bacteriome and metabolome of *Ascaris lumbricoides* in patients

**DOI:** 10.1038/s41598-022-23608-9

**Published:** 2022-11-14

**Authors:** Pavit Klomkliew, Vorthon Sawaswong, Prangwalai Chanchaem, Pattaraporn Nimsamer, Poom Adisakwattana, Orawan Phuphisut, Phornpimon Tipthara, Joel Tarning, Sunchai Payungporn, Onrapak Reamtong

**Affiliations:** 1 Program in Bioinformatics and Computational Biology, Graduate School, Chulalongkorn University, Bangkok, 10330 Thailand; 2 Center of Excellence in Systems Microbiology, Faculty of Medicine, Chulalongkorn University, Bangkok, 10330 Thailand; 3 Department of Helminthology, Faculty of Tropical Medicine, Mahidol University, Bangkok, Thailand; 4 Mahidol Oxford Tropical Medicine Research Unit, Faculty of Tropical Medicine, Mahidol University, Bangkok, Thailand; 5 Centre for Tropical Medicine and Global Health, Nuffield Department of Clinical Medicine, University of Oxford, Oxford, UK; 6 Department of Biochemistry, Faculty of Medicine, Chulalongkorn University, Bangkok, 10330 Thailand; 7 Department of Molecular Tropical Medicine and Genetics, Faculty of Tropical Medicine, Mahidol University, Bangkok, 10400 Thailand

**Keywords:** Molecular biology, Molecular medicine

## Abstract

The most frequent intestinal helminth infections in humans are attributed to *Ascaris lumbricoides*, and there are concerns over the anthelminthic resistance of this species. The gut microbiota has essential roles in host physiology. Therefore, discovering host-parasite–microbiota interactions could help develop alternative helminthiasis treatments. Additionally, these interactions are modulated by functional metabolites that can reveal the mechanisms of infection and disease progression. Thus, we aimed to investigate bacteriomes in the gut of helminths and fecal samples of patients via next-generation sequencing. Our results showed that infection intensity was associated with the bacterial composition of helminth guts but not with the intestinal bacteriome of human hosts. Moreover, the metabolomes of *A. lumbricoides* in the heavy and light ascariasis cases were characterized using ultra-high performance liquid chromatography/time-of-flight mass spectrometry. Increased levels of essential biomolecules, such as amino acids, lipids, and nucleotide precursors, were found in the guts of helminths isolated from heavily infected patients, implying that these metabolites are related to egg production and ascariasis pathogenicity. These findings are the first step towards a more complete understanding of the mechanisms by which the bacteriome of helminth guts affect their colonization and may reveal novel and more effective approaches to parasitic disease therapy.

## Introduction

Ascariasis is a common intestinal helminth infection caused by the large nematodes *Ascaris lumbricoides*, which are among the most common parasites of humans. The parasite was originally widely dispersed in tropical nations with poor sanitation. According to statistics from 2010, an estimated 800 million to 1.2 billion people were infected worldwide^1^. Humans are the most prominent hosts of *A. lumbricoides* due to their oral intake of food contaminated with the nematode eggs. The hatched larvae migrate to the lungs before returning to the stomach and maturing into adult worms in the small intestine. Adult worms that have been sexually fertilized can produce approximately 200,000 eggs per day^2^. Most people with mild ascariasis infections show no symptoms. However, when a roundworm infestation increases in severity, symptoms can become visible, the nature of which depend on the part of the body that is affected. Other signs and symptoms include a persistent cough and bloody mucus, which are due to the larvae’s migration through the bloodstream into the lungs, while adult worms in the small intestine can cause severe abdominal pain and diarrhea^3,4^. Anthelminthic medications such as albendazole (400 mg orally as a single dose), mebendazole (100 mg orally twice daily for three days or 500 mg single dose), and pyrantel pamoate (11 mg/kg orally as a single dose, maximum of 1 g, recommended for pregnancy) are widely used to prevent parasite migration in ascariasis patients^5,6^. However, recent reports on the efficacy of albendazole against large roundworms in Rwandan schoolchildren have raised concerns about anthelminthic resistance in humans^7^. In addition, rapid re-infection with *A. lumbricoides* was observed in Yunnan province, where the prevalence 6 months post-treatment was 83.8%^8^.

At present, metagenomics approaches involve analyzing the entire microbial genome or targeting variable regions of bacterial 16S rRNA genes to characterize microbial groups in diverse communities using next-generation sequencing (NGS), also known as high-throughput sequencing. The parasites can affect the microbial ecosystems in host guts. Helminths that live in the gut lumen can interact with their host microbiota directly through physical touch, chemical compounds, or nutrient competition. Parasites can also affect the microbiota indirectly by altering the host’s physiology and immunity. Despite the fact that host-parasite–microbiota interactions have drawn a lot of academic attention, the outcomes of studies have been inconsistent^9^. Some investigations have discovered that the gut microbiota protects the host from helminths^10^. In contrast, other research has demonstrated that the microbiome aids parasite infections^11^. In addition, understanding the characteristics of host-parasite–microbiota interactions could help with dysbiosis and helminthiasis treatment^12^, and the mechanisms underlying these interactions are currently being unraveled. Similarly, both roundworms and flatworms have been found to have endosymbionts^13^. Filarial parasites such as *Onchocerca volvulus*, *Wuchereria bancrofti*, and *Brugia malayi*, agents of human lymphatic filariasis, are the most well-known examples of nematodes with a mutualistic association with bacteria. The fitness, propagation, and survival of these worms have all been shown to be dependent on endosymbiotic bacteria of the genus *Wolbachia*, which has led to a surge in research into new filaricidal drugs^14^. *Neorickettsia* bacteria have been found in endoparasitic trematodes. These intracellular bacteria live in the reproductive tissues of the worms and are passed vertically to the next generation of parasites via the eggs^13^. Furthermore, horizontal *Neorickettsia* transmission from the fluke to the fluke-infected vertebrate host, where the bacteria infiltrate macrophages and other cell types, is a determinant for the pathogenesis of severe diseases in horses, dogs, and humans^15^.

Until now, host–parasite–microbiota interactions within ascariasis patients have not been investigated, and their roles in pathogenesis remain unclear. To better understand the function of the parasite-associated microbiome in helminthiasis pathophysiology, the bacterial profiles of the feces of patients with heavy, moderate, and light *A. lumbricoides* infections were compared with those of healthy controls in this study. The microbiota of large roundworm intestines from patients of different infection statuses were also investigated using NGS. In addition, comparative metabolomics were applied to the *A. lumbricoides* intestines from mild- and high-intensity infections with the aim of gathering information on the pathways contributing to infection pathogenicity. This fundamental understanding may reveal parasite vulnerabilities, paving the way to the development of novel control strategies.

## Results

### Data sequencing output

After high-throughput sequencing of the 16S rRNA gene, a total of 2,343,769 raw reads for human feces and two intestinal tissues samples from *A. lumbricoides* (anterior and posterior gut) were obtained for analysis, with an average of 34,467 sequences per sample. The raw reads were retained after quality filtering, and the bacterial taxa were classified into operational taxonomic units (OTUs). The sequence reads were used to estimate whether there was sufficient sequence coverage to reliably describe all samples by rarefaction analysis (Supplementary Fig. S1). An overview of the data sequencing output for each group is provided in Table 1.

**Table 1:** Sequencing summary of 16S bacteriome in each group.

Sample group	n	Total raw reads*	Total retained reads*	% Classified
Intestinal tissue of worm	38	32,806 ± 21,490	32,746 ± 21,425	91.49
Human feces	30	36,571 ± 23,599	36,451 ± 23,525	93.85

*Data shown in table were presented as mean ± SD.

### Gut bacteriome profile and diversity in humans

Based on a Mann–Whitney U test of the Chao1 and Shannon indexes, there were no significant differences between the gut bacteriomes of ascariasis patients and uninfected human hosts (Supplementary Fig. S2A-B). The fecal bacteriome communities of uninfected and infected humans could not be clearly distinguished using the beta diversity based on Bray–Curtis distance (PERMANOVA, *P* < 0.001) in Supplementary Fig. S2C. The most abundant phylum was Firmicutes, followed by Bacteroidetes and Proteobacteria. At the genus level, the most abundant bacteria in the human gut were *Prevotella*, which also showed significant differences in abundance (Mann–Whitney U test, *P* < 0.05) between ascariasis patients (16.6%) and uninfected human hosts (13.1%) among the top 24 most abundant taxa, as showed in Supplementary Fig. S2D. Additionally, the relative abundances of members of the Ruminococcaceae and Lachnospiraceae families were decreased in the gut of ascariasis patients, while *Streptococcus* was more abundant in uninfected patients. However, *Streptococcus* was found in small number of uninfected human hosts.

### Gut bacteriome profile and diversity in *A. lumbricoides*

**Figure 1:**
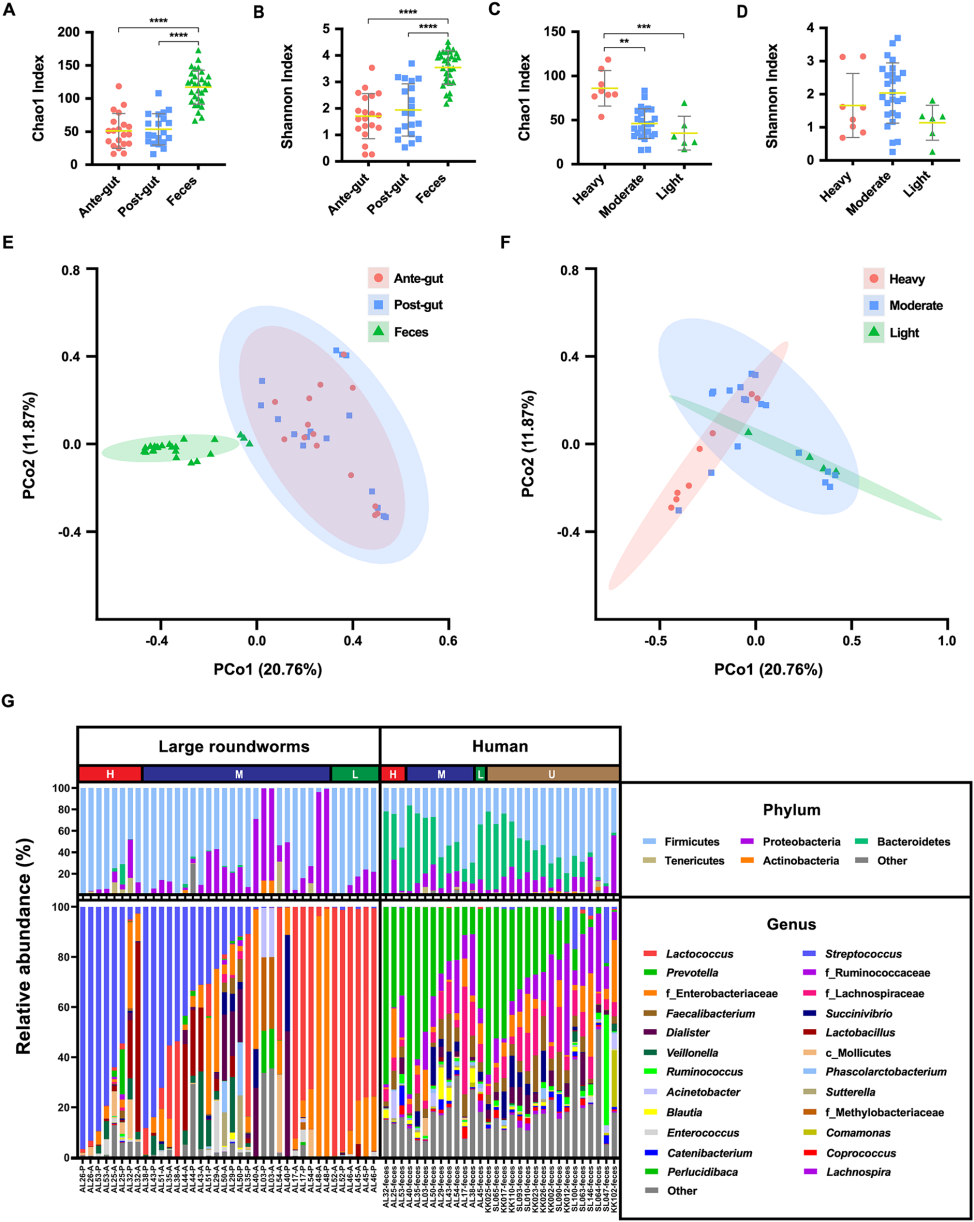
Gut bacteriome diversity and composition in *A. lumbricoides* and humans. The alpha diversity comparison of bacteriome in (**A**, **B**) type of samples, and (**C**, **D**) infection intensity of patients calculated by Chao1 index and Shannon index were shown as scatter plots with the error bars representing the standard deviation and performed statistically significant difference by Kruskal–Wallis test (**P < 0.01, ***P < 0.001, **** *P* < 0.0001). The beta diversity of bacteriome between (**E**) type of samples and (**F**) infection intensity of patients was presented by principal coordinate analysis (PCoA) plots based on Bray–Curtis distance. (**G**) The stacked plot showed relative abundances of gut bacteriome at the phylum and genus levels in the infection intensity of patients (H = heavy infected, M = moderate infected, L = light infected and U = uninfected) assessed by high throughput sequencing on the 16S rRNA gene.

The microbiomes of the anterior and posterior gut sections of *A. lumbricoides* guts were analyzed by NGS. To illustrate the richness and evenness of bacterial species based on their relative abundances, alpha diversity with Chao1 index and Shannon indexes was performed. The bacterial diversities in the anterior and posterior guts of large roundworms were not significantly different in the Wilcoxon matched-pairs signed rank test (Fig. 1A-B). The Bray–Curtis distance between the bacteriomes in the anterior and posterior guts of large roundworms showed a high degree of clustering, with identity at a 95% confidence interval (Fig. 1E). The relative abundances of gut bacteria in *A. lumbricoides* were generated at the phylum and genus levels and grouped across different gut sections (Supplementary Fig. S3). It was obvious that the phyla Firmicutes (71.5%) and Proteobacteria (23.5%) dominated the gut bacteriome, and at the genus level, *Streptococcus* (27.9%) and *Lactococcus* (26.4%) were the most prevalent gut bacteria of large roundworms.

### Comparison of human and *A. lumbricoides* gut bacteriomes

Alpha diversity based on Kruskal–Wallis tests of the Chao1 and Shannon indexes showed that the fecal bacteriome of humans had significantly higher diversity than the intestinal bacteriome of *A. lumbricoides* (*P* < 0.05), as shown in Fig. 1A-B. The Chao1 richness of heavily infected patients was significantly higher than the other two patient groups (Fig. 1C), while the Shannon index showed no significant difference with infection intensity (Fig. 1D). The Bray–Curtis distance greatly separated the gut bacteriomes of human and large roundworms, while those in the anterior and posterior guts of *A. lumbricoides* were very similar (Fig. 1E). In addition, the microbiomes of heavily infected patients were separate from those of moderately and lightly infected patients (Fig. 1F).

The relative abundances of the gut bacteriomes in humans and *A. lumbricoides* were compared at the phylum and genus levels among the different degrees of ascariasis severity (Fig. 1G). The overall gut bacteriome compositions in humans and *A. lumbricoides* were dominated by Firmicutes at the phylum level, with a total average relative abundance of over 62% in all groups. Bacteroidetes (34.3%) dominated the guts of humans, whereas Proteobacteria (23.5%) predominated in *A. lumbricoides*. However, the comparative results showed there were no differences in the bacterial phyla in the gut bacteriomes of humans or *A. lumbricoides* with infection intensity. As with the bacterial genera of *A. lumbricoides*, the predominant bacterial taxa differed with the infection status of the hosts. In heavily infected people, the most abundant genus was *Streptococcus* (12.8%), while in lightly infected hosts, *Lactococcus* dominated (13.5%). Moreover, *Lactococcus* (12.7%) and *Streptococcus* (15.1%) were the most commonly observed bacterial genera in the moderately infected individuals. Contrary to the observations of the gut bacteria in large roundworms, *Lactococcus* and *Streptococcus* were rarely observed in human gut bacteriomes. Intriguingly, the *Prevotella* genus and Ruminococcaceae and Lachnospiraceae families predominated the gut bacteriomes of ascariasis human hosts. Notably, the gut bacterial profiles of humans did not differ with infection intensity.

### Differential abundance of gut bacteriome in *A. lumbricoides* with infection intensity

**Figure 2:**
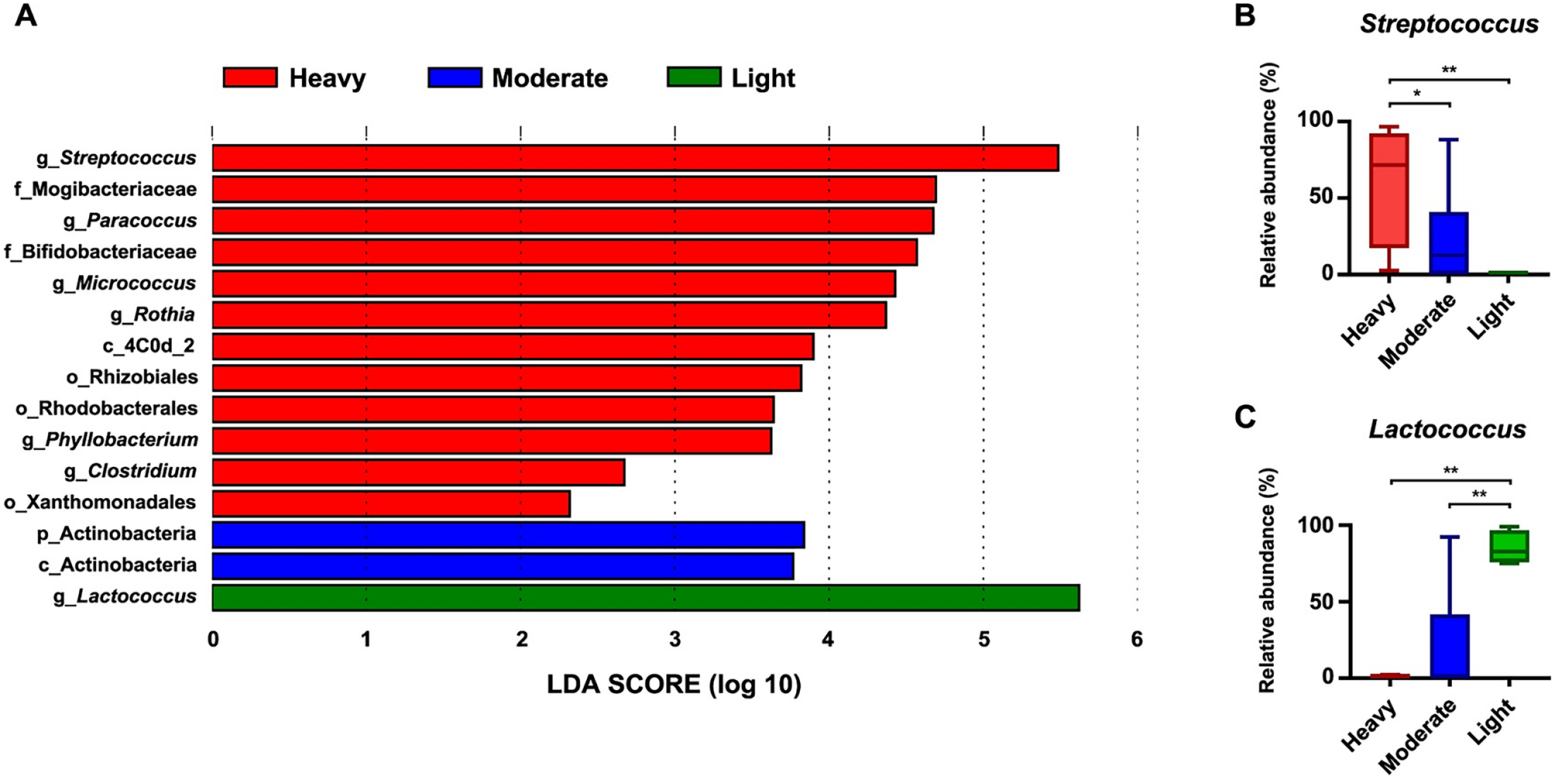
The differential abundance of bacterial taxa in *A. lumbricoides* among the infection intensity based on Linear discriminant analysis Effect Size (LEfSe) analysis. (**A**) LDA score of significantly enriched bacteria among groups. Based on LDA score > 5, The relative abundance of (**B**) *Streptococcus* and (**C**) *Lactococcus* were compared among infection intensity. Statistical analysis was performed by Kruskal–Wallis test (*P < 0.1, **P < 0.01).

To gain more information on the differential abundance of gut bacteriome species*,* LEfSe analysis of gut microbiota in *A. lumbricoides* was performed for the different infection intensities, and the results are shown in Fig. 2A. *Streptococcus* (Fig. 2B) and *Lactococcus* (Fig. 2C) were significantly enriched in heavily and lightly infected patients, respectively, based on their Linear discriminant analysis (LDA) scores (> 5). Moreover, Actinobacteria species showed a significantly higher abundance in moderately infected patients, whereas *Paracoccus*, *Micrococcus,* and *Rothia* were enriched in the bacteriomes of heavily infected patients*.*

### Metabolomic analysis of *A. lumbricoides* guts in patients with heavy and light infection intensities

**Figure 3:**
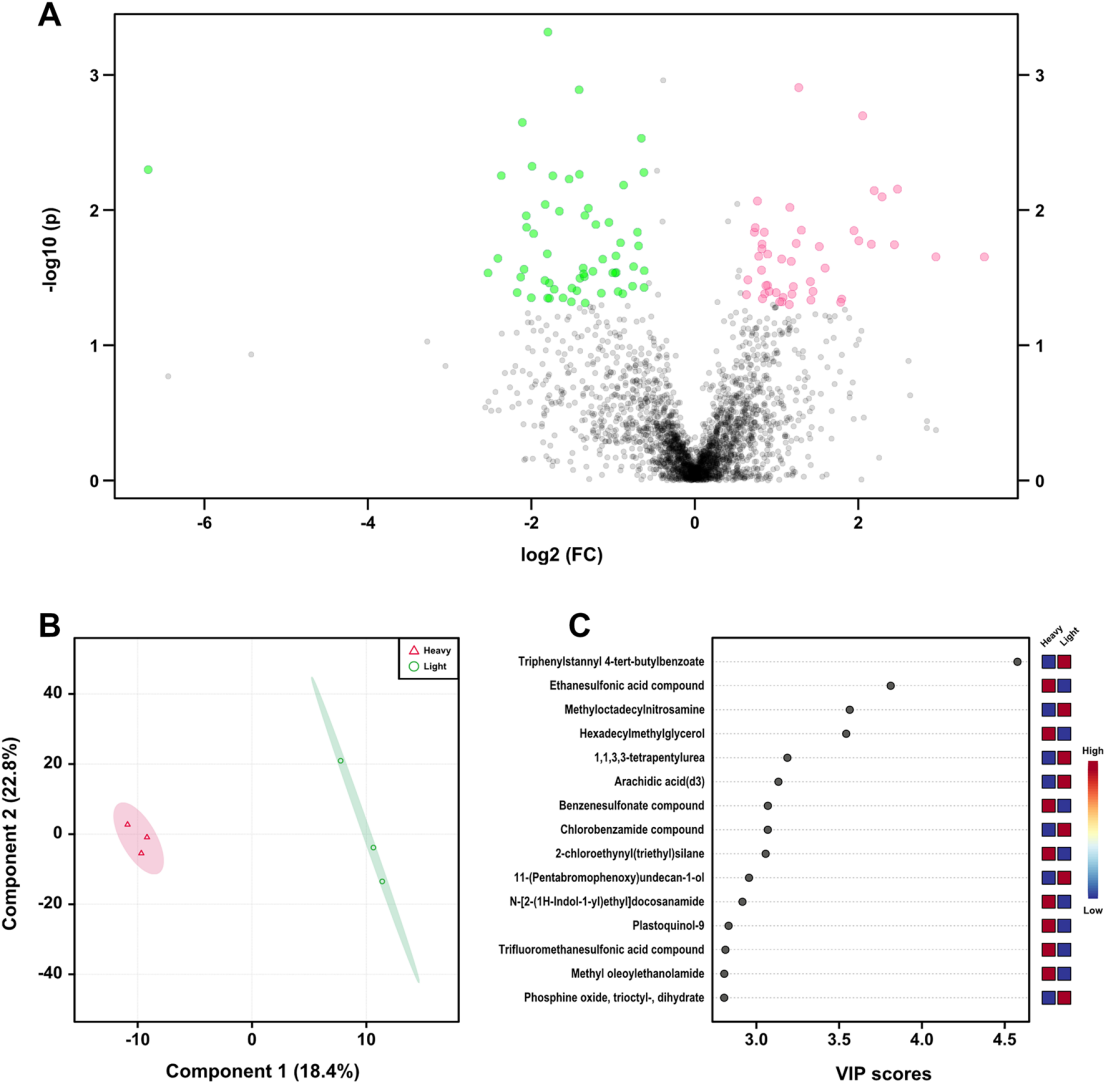
Gut metabolomics for quantification of metabolites based on positive ion mode between the heavy and light infected ascariasis. (**A**) Volcano plot showing the statistical significance and fold change of metabolites in heavy infected (red) compared to light infected (green) of ascariasis (*P* < 0.05, Fold change ≥ 1.5). (**B**) Score plots of partial least squares discriminant (PLS-DA) analysis of metabolome from heavy and light infected samples. The color around each group represents the 95% confidence intervals. (**C**) The plot representing 15 m/z features with top variable importance in projection (VIP) score calculated based on PLS-DA analysis.

Since *Streptococcus* and *Lactococcus* were dominant in *A. lumbricoides* guts in heavy and light infection intensity patients, an untargeted metabolomics approach was applied to analyze the metabolite profiles of the parasite guts. A total of 4998 m/z features were identified in both the positive and negative ion modes. The results, illustrated in the volcano plots in Fig. 3A and Supplementary Fig. S4A, revealed the presence of several positively and negatively charged ions (fold change > 1.5). Of these, 91 and 121 m/z features were significantly increased (*P* < 0.05) in the heavily and lightly infected ascariasis groups, respectively. Partial least square discrimination analysis (PLS-DA) analysis was performed to visualize separation trends among groups based on the abundance of differentially expressed metabolites. There were significant differences in the compositions of gut metabolites identified from the positive and negative modes in the heavy and light ascariasis cases (Fig. 3B and Supplementary Fig. S4B).

The m/z features with highest variable importance in projection (VIP) scores, calculated based on PLS-DA analysis, were identified by accurate mass measurement using the Metlin database (Supplementary Table S1). Several specific metabolites, such as ethanesulfonic acid compound, hexadecyl methyl glycerol, benzenesulfonate compound, 2-chloroethynyl(triethyl)silane, hexyldioxodecyl methyl tyrosinate, apicidin, and aripiprazole lauroxil, were significantly increased in heavy ascariasis. However, triphenylstannyl 4-tert-butylbenzoate, methyloctadecylnitrosamine, 1,1,3,3-tetrapentylurea, arachidic acid(d3), chlorobenzamide compound, 1-phenylpyrazole;titanium(3 +), HR1917, haploside A, benzenesulfonamide compound, avicularin, 8-methylthiooctyl glucosinolate, malvidin 3-o-glucoside, acetamide compound, flutropium bromide, trifluoroiodomethane, and diazoacetic acid tetradecyl ester were enriched in light ascariasis (Fig. 3C and Supplementary Fig. S4C).

### Different metabolic pathways in the gut of *A. lumbricoides* in heavily and lightly infected ascariasis patients

**Figure 4:**
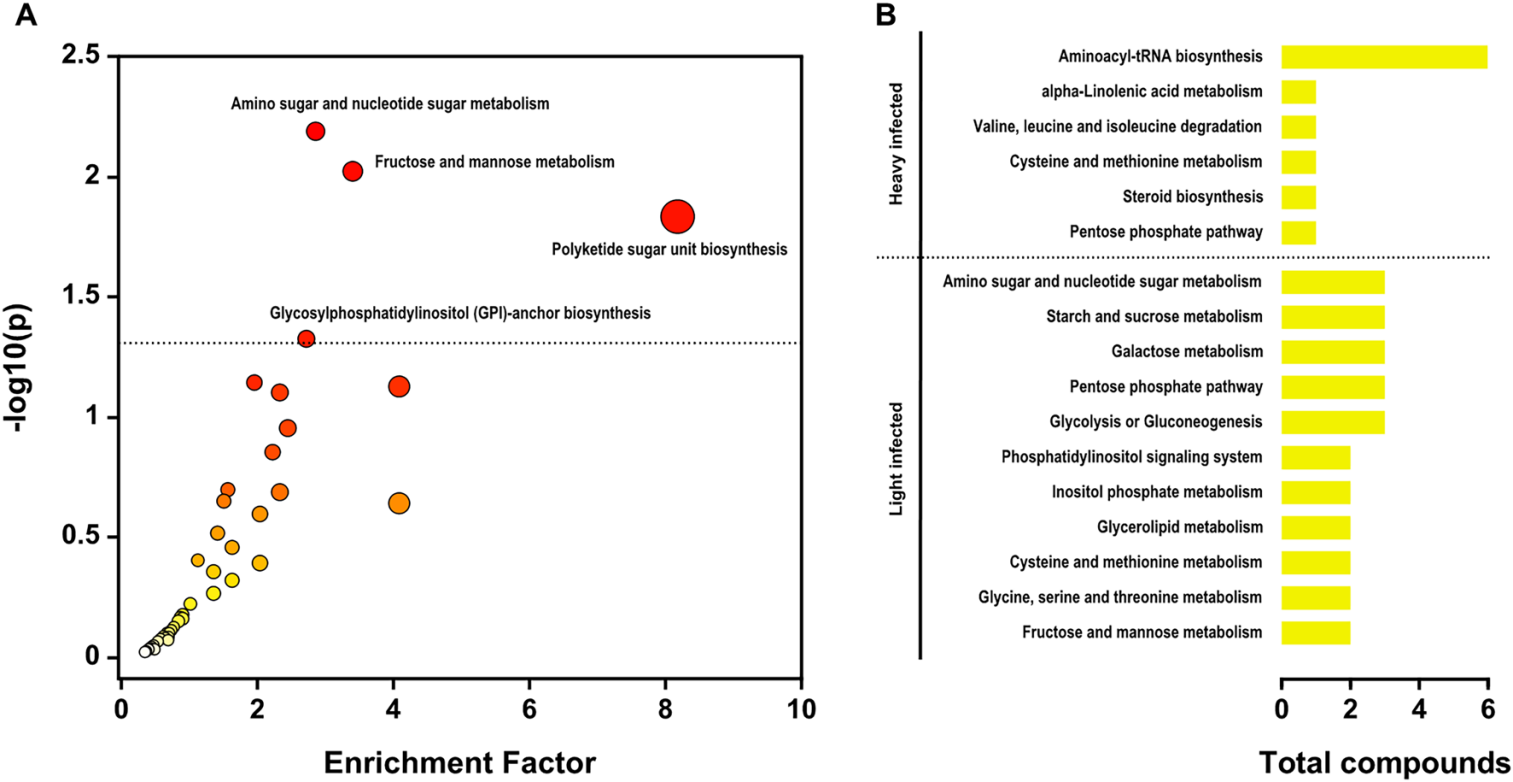
Metabolite annotation and pathway analysis using Mummichog. (**A**) Pathway enrichment is based on altered metabolites in both the heavy and light infected ascariasis groups. (**B**) Pathway enrichment and proportion of compound metabolites were shown separately in the heavy and light infected ascariasis.

Pathway enrichment based on the altered metabolites in both the heavily and lightly infected ascariasis groups were analyzed using Mummichog. Metabolite annotation and pathway enrichment analysis identified 52 significant pathways (*P* < 0.001) and at least three annotated metabolites. The 52 pathways, including amino sugar and nucleotide sugar metabolism, fructose and mannose metabolism, polyketide sugar unit biosynthesis, and glycosylphosphatidylinositol-anchor biosynthesis, were significantly (*P* < 0.05) enriched in both heavily and lightly infected ascariasis cases (Fig. 4A and Supplementary Table S2). Differentially accumulated analysis and the 1.5-fold difference in m/z features from heavy and light ascariasis were evaluated by Mummichog (*P* < 0.005) and error tolerance (± 5 PPM). In the heavily infected group, the most abundant metabolites were related to aminoacyl-tRNA biosynthesis; alpha-linolenic acid metabolism; Val, Leu, and Ile degradation; Cys and Met metabolism; steroid biosynthesis; and the pentose phosphate pathway (Fig. 4B). In contrast, the lightly infected group showed a higher abundance of metabolites related to amino sugar and nucleotide sugar metabolism; starch and sucrose metabolism; galactose metabolism; the pentose phosphate pathway; glycolysis or gluconeogenesis; the phosphatidylinositol signaling system; inositol phosphate metabolism; glycerolipid metabolism; Gly, Ser, and Thr metabolism, and fructose and mannose metabolism (Fig. 4B).

### Multi-omics integration network analysis

**Figure 5:**
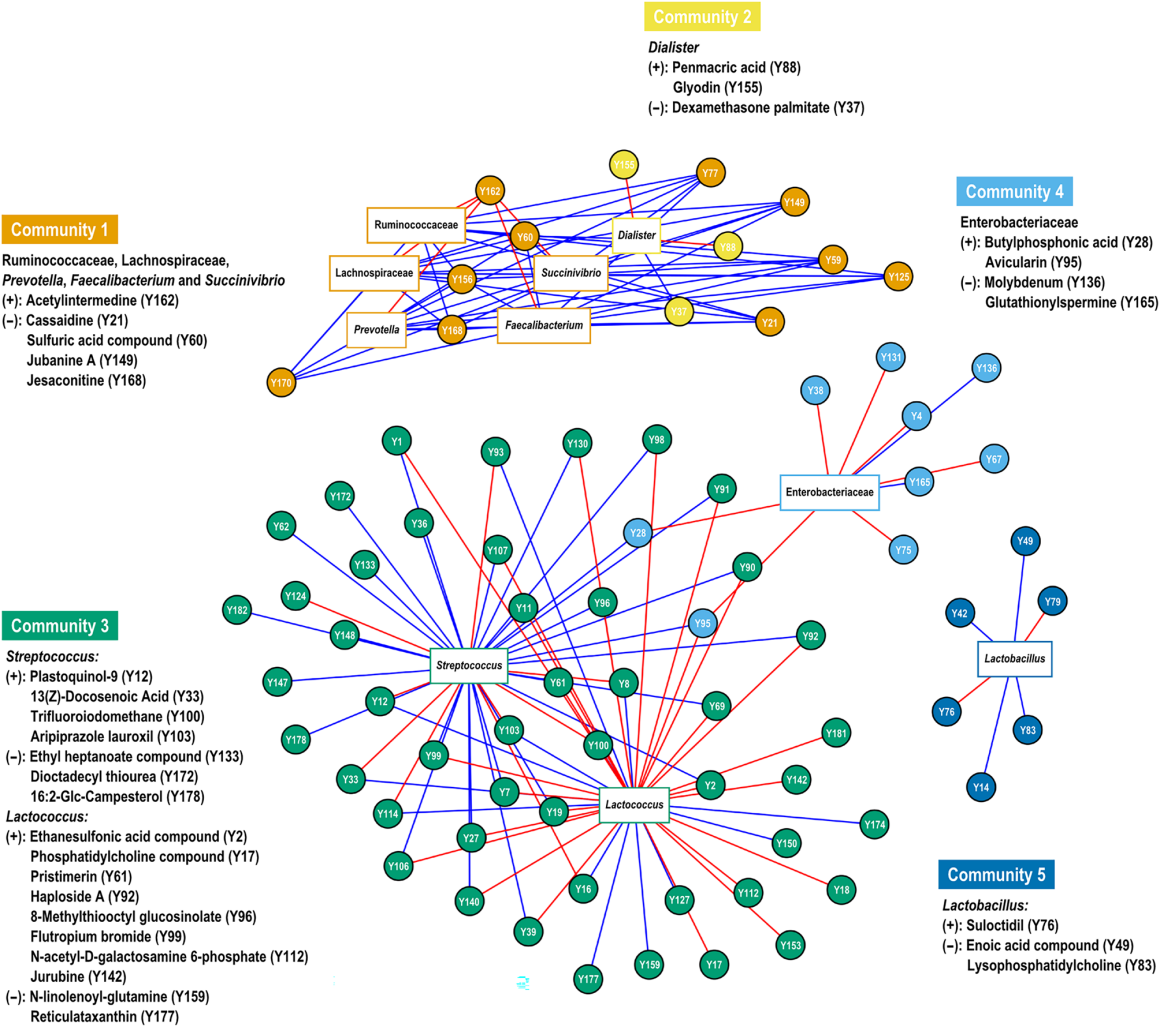
Multi-omics integration performed with xMWAS identified five communities. Encode name of metabolite features and bacterial are shown in circles and squares, respectively. Links indicate pairwise Pearson’s correlations (|*r*|> 0.40). Red links indicate a positive correlation, whereas blue links indicate a negative correlation.

Integrative network analysis was carried out to establish the relationships between the relative abundances of bacteria and the top 200 metabolites based on the highest VIP score in the positive and negative modes. The network comprised five clusters with 10 bacteria genera/families that were statistically correlated with metabolite features (|*r*|> 0.4 and *P* < 0.05). These bacteria included *Lactococcus* (39 features), *Streptococcus* (38 features), Lachnospiraceae (10 features), Ruminococcaceae (10 features), *Prevotella* (10 features), *Faecalibacterium* (10 features), *Succinivibrio* (10 features), Enterobacteriaceae (9 features), *Lactobacillus* (6 features), and *Dialister* (4 features) (Fig. 5 and Supplementary Table S3). In Community 1, *Prevotella*, *Faecalibacterium*, *Succinivibrio*, Ruminococcaceae, and Lachnospiraceae were negatively correlated with cassaidine, jubanine A, jesaconitine, and sulfuric acid compounds were specifically positively correlated with acetylintermedine. In Community 2, the compounds glyodin and penmacric acid were positively correlated with *Dialister*, but dexamethasone palmitate was negatively correlated with *Dialister.* In Community 3, *Streptococcus* showed a negative correlation with ethyl heptanoate, dioctadecyl thiourea, and 16:2-Glc-campesterol compounds, whereas *Lactococcus* showed a positive correlation with *N*-acetyl-*D*-galactosamine 6-phosphate, jurubine, and phosphatidylcholine compounds. Additionally, some metabolites (trifluoroiodomethane, aripiprazole lauroxil, plastoquinol-9, and 13(*Z*)-docosenoic acid) were negatively correlated with *Lactococcus* but positively correlated with *Streptococcus*. Similarly, the m/z features of haploside A, pristimerin, ethanesulfonic acid compound, 8-methylthiooctyl glucosinolate, and flutropium bromide were positively correlated with *Lactococcus* but negatively correlated with *Streptococcus*. In the overlapping Communities 3 and 4, avicularin and butylphosphonic acid compounds were negatively correlated with *Streptococcus*, but these compounds were positively correlated with *Lactococcus* bacteria and Enterobacteriaceae. Additionally, some compounds of molybdenum and glutathionylspermine showed a negative correlation with Enterobacteriaceae. Finally, *Lactobacillus* was negatively correlated with enoic acid and lysophosphatidylcholine compounds, whereas they were positively correlated with suloctidil, as part of Community 5.

## Discussion

Ascariasis is a common intestinal helminth infection caused by *A. lumbricoides* that can impact the underlying mechanisms of the host immune system^16^. However, there is limited information on the gut microbiome of *A. lumbricoides*-infected patients, and we have a distinct lack of knowledge of the gut microbiome of *A. lumbricoides*. This is the first report characterizing the microbial heterogeneity of human and large roundworm microbiomes. We utilized the microbiomic and metabolomic signatures of human and large roundworms guts to answer fundamental questions regarding host-parasite interactions in ascariasis patients.

In this study,the beta diversity based on Bray–Curtis distance showed that fecal bacteriome communities were significantly separated between uninfected and infected humans. This result corresponds to findings on *A. suum* infection: infection with this nematode was demonstrated to alter the host microbiome and affect 58 metabolic pathways^17^.

In our cohort, we found that both human and *A. lumbricoides* gut microbiomes were dominated by bacteria of the phylum Firmicutes. In addition, Bacteroidetes and Proteobacteria dominated the microbiomes of humans and *A. lumbricoides*, respectively. A balanced ratio between Firmicutes and Bacteroidetes plays a crucial role in several aspects of human health, including metabolism and inflammation, and dysbiosis of these bacteria is associated with the progression of diseases^18^. An imbalanced microbiome is frequently caused by a continuous increase in the abundance of the phylum Proteobacteria. This phylum is found in only a small percentage of the natural human gut flora, and its increased prevalence has been linked to decreased populations of Firmicutes, which leads to the progression of colonic inflammation and metabolic disorders^19^. Because Proteobacteria was the dominant family in the *A. lumbricoides* gut, these bacteria might be one of the variables disturbing the function of the human gut. Proteobacteria species have also been found in the *Trichuris muris* microbiome^20^. Several Proteobacteria are facultative anaerobes; they may use any available oxygen within the nematode intestinal tract, making conditions more conducive for the nematodal microbiota’s obligatory anaerobic members. At the genus level, a significant proportion of bacteria in the gut of ascariasis patients were identified as species of *Prevotella*. This was also discovered in villagers from Liberia and Indonesia who were infected by soil-transmitted helminths^21^. *Prevotella* is known to play a key role in carbohydrate metabolism, and an increase in these bacteria seems to be associated with *Schistosoma haematobium* infection^22^. This result is supported by previous research on the effects of helminth infections on the gut bacteriome composition in humans^9,23,24^.

When *A. lumbricoides* gut microbiomes in humans with different infection intensities were compared, we found *Streptococcus* to be the most common bacteria in the *A. lumbricoides* gut in high-intensity infections, whereas *Lactococcus* dominated in *A. lumbricoides* guts in low-intensity infections. The genera *Streptococcus* and *Lactococcus* are involved in the fermentation of sugars, and both bacteria are known to have health-promoting effects^25^. *Streptococcus* can significantly reduce mucosal pro-inflammatory activity in the human colon^26^. This evidence indicates that infection intensity may be responsible for significant changes in the gut bacteriome of *A. lumbricoides*.

Pathway analysis was performed to reveal the important mechanisms in *A. lumbricoides* gut in heavy and light infection intensities. In *A. lumbricoides* guts at heavy infection intensity, metabolites related to aminoacyl-tRNA biosynthesis; alpha-linolenic acid metabolism; Val, Leu, and Ile degradation; Cys and Met metabolism; steroid biosynthesis; and the pentose phosphate pathway were increased. Aminoacyl-tRNA synthetases (ARSs) play important roles in protein synthesis by linking amino acids to their cognate transfer RNAs. ARSs are engaged in immune cell maturation, transcription, activation, and recruitment, and hence play a critical role in immune cell development^27,28^. Therefore, the increase in aminoacyl-tRNA biosynthesis in the *A. lumbricoides* gut might enhance the immunity of this large roundworm. Apart from ARS, several essential biomolecule metabolic processes, e.g., amino acid, sterol, and nucleotide pathways, were also enhanced in *A. lumbricoides* guts in heavy infections. The pentose phosphate pathway generates NADPH and pentoses as well as ribose 5-phosphate—a precursor for the synthesis of nucleotides^29^. Pathways relating to the production of important biomolecules, including alpha-linolenic acid metabolism; Val, Leu, and Ile degradation; Cys and Met metabolism; and steroid biosynthesis, also increased in *A. lumbricoides* guts in high-intensity infections. This might indicate that *A. lumbricoides* in heavy infections obtain more amino acids, nucleotide precursors, lipids, and essential biomolecules by enhancing these metabolic processes, possibly leading more egg production, which increases incidences of ascariasis. In contrast, the metabolites increased in *A. lumbricoides* guts in low-intensity infections are mainly involved in carbohydrate metabolism, including amino sugar and nucleotide sugar metabolism, starch and sucrose metabolism, galactose metabolism glycolysis or gluconeogenesis, phosphatidylinositol signaling system, inositol phosphate metabolism, and fructose and mannose metabolism. This increase in carbohydrate metabolism might contribute to energy supply rather than egg production.

According to the integration network analysis of the Community 3, *Streptococcus* abundance positively correlated with trifluoroiodomethane, aripiprazole lauroxil, plastoquinol-9, and 13(Z)-docosenoic acid. There is a lack of information on the functions of most of these metabolites in parasites, and only general information on plastoquinol-9 and 13(Z)-docosenoic acid is available. Plastoquinone is an isoprenoid quinone molecule involved in interactions between photosystem II and cytochrome b6f. in the complex electron transport chain^30^; thus, this metabolite might play role in electron transport in *A. lumbricoides* gut. In vivo*,* 13(Z)-docosenoic acid could be metabolized to oleic acid^31^, and the C-1 amide of docosenoic acid has been discovered to be a anandamide-related neurotransmitter^32^. Therefore, 13(Z)-docosenoic acid may be involved in neurotransmitter production in *A. lumbricoides*. The abundance of *Lactococcus* showed positive correlations with N-acetyl-D-galactosamine 6-phosphate, jurubine, phosphatidylcholine compound, haploside A, pristimerin, ethanesulfonic acid compound, 8-methylthiooctyl glucosinolate, and flutropium bromide. There is also a limited amount of information on most of these metabolites in parasites, and more experiments need to be performed to understand their biological roles. Jurubine, which has been found in the root-knot nematode^33^, is an alkaloid with antioxidant properties^34^. Pristimerin displays several pharmacological effects, such as anti-cancer, antioxidant, anti-inflammatory actions^35^. Therefore, jurubine and pristimerin may play antioxidant and anti-inflammation roles in the *A. lumbricoides* gut.

Our study had some limitations, including a lack of information on the human volunteers’ food consumption, antibiotic uptake, and bacterial infection history. Moreover, the abundances of bacteria were not validated by quantitative PCR due to insufficient remaining samples in this study. However, the findings provide insightful data that further advance our understanding of microbiome–metabolome interactions between humans and large roundworms. Moreover, no previous studies have shown a direct effect of ascariasis on the bacterial composition in large roundworm guts. Although we found correlations between metabolite molecules and bacterial genera in large roundworms, these bacteriome–metabolome correlations could not be accurately predicted in *A. lumbricoides*, and targeted LC–MS analysis was not performed to validate the metabolites. Nevertheless, these discoveries are hypothesis-generating and should be substantiated in future mechanistic research.

In conclusion, this study demonstrated that infection intensity was associated with the gut bacterial microbiota of *A. lumbricoides* but not human hosts. In addition, the increased levels of essential biomolecules, such as amino acids, lipids, and nucleotide precursors, were observed in the guts of *A. lumbricoides* isolated from patients with heavy infection intensity, implying that these metabolites might be associated with egg production and ascariasis pathogenicity. The discovery of the gut bacterial microbiota and metabolite profile of *A. lumbricoides* may be useful for development of novel and more effective approaches for parasitic disease therapy in the future.

## Methods

### Study cohort and characteristics subjects

This procedure was approved by the Ethics Committee of the Faculty of Tropical Medicine, Mahidol University (MUTM 2021–020-01). All methods were performed in accordance with the relevant guidelines and regulations. All participants provided written consent to participate in the study and for the use of anonymous quotations. Participants with *A. lumbricoides* infections assessed by the Kato–Katz technique were received a single oral dose of albendazole in accordance with WHO recommendations^1^. The stools were collected daily for four days and *A. lumbricoides* adult worms were recovered from the stool sample and rinsed several times with sterile 0.85% normal saline solution (NSS) to remove fecal contamination. The samples were kept at − 80 °C until used. Feces samples (n = 30) and large roundworm samples (n = 19) were collected from school-age children and young adults at Ban Mae Salid Luang and Gre Key Village, Thailand (Supplementary Table S4). Each fecal human sample was examined for *A. lumbricoides* eggs using Kato-Katz smears with two independent slides by two independent microscopists. The number of *Ascaris* eggs was counted, and the mean number of *Ascaris* eggs per gram of feces was calculated and used to estimate the intensity of the ascariasis. The infection intensity was categorized as light when there were fewer than 5000 large roundworm eggs per gram (EPG) of feces, moderate when there were 5000–50,000 EPG, and heavy when there were more than 50,000 EPG^36,37^ before anthelminthic treatment under the supervision of a physician by the laboratory of the Department of Helminthology, Mahidol University. In human hosts who were found to be infected, the large roundworms were expelled, adult worm samples were collected for use in experiments, and worm intestinal tissue was dissected into two sections (anterior and posterior gut) to compare the bacteriomes in each niche. The feces and intestinal tissues of worms were preserved in a microtube and stored in lysis buffer at − 80 °C before DNA extraction. The human hosts were divided into categories, including the village of origin, sex, age, and status of ascariasis, for further testing, as shown in Table 2.

**Table 2:** The characteristics of human hosts from Ban Mae Salid Luang (BL) and Gre Key (GK) village.

Category		Village of origin	
		BL	GK
Total number of human hosts		18	12
Sex of human hosts	Male	11	5
	Female	7	7
Age of human hosts	Child and adolescent (2–18 years)	13	6
	Young adult (19–24 years)	5	6
Total number of infection intensity	Heavy infected	2	1
	Moderate infected	7	2
	Light infected	NA	1
	Uninfected	9	8

*NA   not applicable.

### Microbiome profiling by metagenomic approach

#### Total DNA extraction

The individual large roundworm was homogenized by high-speed shaking at 50 Hz for 5 min in plastic tubes with lysis buffer and small stainless beads using TissueLyser LT (Qiagen, Germany). The genomic DNA was extracted from human feces and intestinal tissue of large roundworm samples using the GenUP gDNA extraction kit (Biotechrabbit, Germany) following the manufacturer’s recommendations after removal of small amounts of debris by centrifugation at 13,000 rpm for 5 min. The concentrations and quality of DNA were measured using a NanoPhotometer C40 (Implen, Germany).

#### 16S rRNA library amplification and Illumina sequencing

The hypervariable regions of the bacterial 16S rRNA gene were amplified using semi-nested PCR with a pair of primers, 16S V4_515F (5′-CACGGTCGKCGGCGCCATT-3′) and 16S V4_806R (5′-GGACTACHVGGGTWTCTAAT-3′), in the first amplification and using the dual-index (8 × 12) primer set (Illumina, USA) for DNA library preparation in the second PCR. Following a previous report^38^, the primers were modified by adding a phasing spacer and specific adaptor sequences to their 5′ ends. In a total volume of 20 µL, the PCR reactions contained 50 ng of DNA template, 0.2 mM dNTPs, 0.25 M of each primer, 1 × Phusion green HF Buffer, and 0.02 U of Phusion DNA polymerase (Thermo Fisher Scientific, USA). The PCR was performed on a thermocycler (Qiagen, Hilden, Germany) with following conditions: initial denaturation at 98 °C for 30 s, 25 cycles of amplification (denaturation at 98 °C for 10 s, annealing at 55 °C for 30 s, and extension at 72 °C for 45 s), and a final extension at 72 °C for 10 min. The second PCR was performed with the same thermocycling protocol as the first, except the annealing temperature was adjusted to 60 °C. After that, the DNA library was purified using a QIAquick gel extraction kit (Qiagen, Germany) and evaluated with 2% agarose gel electrophoresis. The concentration of the library was measured using the KAPA library quantification kits (KAPA Biosystems, USA), and the library was pooled to equal concentrations and paired-end sequenced (2 × 250 bp) on the Illumina MiSeq platform (Illumina, USA) with 20% PhiX control.

#### Bioinformatic analyses

The raw sequences data (FASTQ files) were demultiplexed by MiSeq reporter software (version 2.6.2.3), and the FASTQ files were analyzed with the QIIME2 pipeline (version 2019.7)^39^. The merged sequences of paired-end reads were trimmed of Illumina adapters, and low-quality score (< Q30) reads were removed. The filtered reads were deduplicated and clustered for OTUs with a 97% similarity by VSEARCH^40^, and the chimeric reads were removed with the UCHIME algorithm^41^. Finally, the filtered reads were used for taxonomic classification of the bacteria based on the Greengenes database (version 13.5) (https://greengenes.secondgenome.com/). Alpha diversity, beta diversity, and rarefaction analysis were investigated based on QIIME2 pluginsThe statistical analysis of alpha diversity (Chao1 and Shannon indexes) were calculated by Mann–Whitney U test for non-parametric test comparison between two groups and Kruskal–Wallis test for non-parametric test comparison among several groups using GraphPad Prism (version 8.0)^42^. The differential bacteriome composition between samples was analyzed based on the Linear discriminant analysis Effect Size (LEfSe) method, which was set for *P* < 0.01 (Kruskal–Wallis test) and an LDA score threshold of > 2.0^43^ in the Galaxy server (https://huttenhower.sph.harvard.edu/galaxy/).

### Metabolic pathway by metabolomic approach

#### Metabolite extraction

The large roundworms' gut samples (approximately 6 mg) from heavy or light ascariasis patients were transferred into 1.5 mL microcentrifuge tubes and individually homogenized with 500 μL 100% methanol by Dounce homogenizer. The tubes were then snap-frozen in liquid nitrogen and thawed before centrifuged at 2800 rpm for 1 min at 4 °C. The supernatant was collected and transferred into a new tube, whereas the pellet was extracted again as mentioned above. In addition, the pellet was resuspended in 250 μL of deionized water followed by snap-freezing in liquid nitrogen and thawing. The supernatant was obtained by centrifugation at 17,000 rpm for 1 min at 4 °C and then pooled into the same tube with the supernatant obtained from previous steps. The pooled supernatants were centrifuged at 17,000 rpm for 1 min at 4 °C to remove the remaining debris. The clear supernatant was transferred to a new tube and dried under high pressure in a speed vacuum (Tomy Digital Biology, Japan).

#### Metabolite identification by mass spectrometry

We employed ultra-high performance liquid chromatography (UHPLC; Agilent 1260 Quaternary pump, Agilent 1260 High-Performance Autosampler, and Agilent 1290 Thermostatted Column Compartment SL, Agilent Technologies) coupled to a quadrupole time-of-flight mass spectrometer (Q-TOF–MS) (TripleTOF 5600 + , SCIEX, US) with electrospray ionization (ESI) using a DuoSpray ion source. The mobile phase system for UHPLC separation was water containing 0.1% formic acid (mobile phase A) and acetonitrile containing 0.1% formic acid (mobile phase B). The metabolite pellet was reconstituted in 200 μL of mobile phase A: B at a ratio of 50:50 (vol/vol) and transferred to liquid chromatography (LC) vial for injection. LC vials were kept in the auto-sampler at 6 °C during the analysis. Five microliters of sample were injected onto a C18 reversed-phase column (ACQUITY UPLC HSST3, 2.1 × 100 mm, 1.8 µM, Waters) protected by a pre-column (ACQUITY UPLC HSST3, 2.1 × 5 mm, 1.8 µM, Waters) for separation by UHPLC at a flow rate of 0.3 mL/min at 40 °C. The UHPLC elution gradient was started at 5% mobile phase B for 2.0 min (0.0–2.0 min), 5%–60% B for 0.5 min (2.0–2.5 min), 60%–80% B for 1.5 min (2.5–4.0 min), 80%–100% B for 8.0 min (4.0–12.0 min), 100% B for 5 min (12.0–7.0 min), 100%–5% B for 0.1 min (17.0–17.1 min), and 5% B for 2.9 min (17.1–20.0 min). The UHPLC-Q-TOF–MS system, mass ion chromatogram, and mass spectra were acquired by Analyst Software version 1.7 (SCIEX). The Q-TOF–MS was operated in positive (+ ESI) and negative (-ESI) electrospray ionization modes. Ion source gas 1 was set at 45 psi, ion source gas 2 at 40 psi, curtain gas at 30 psi, and source temperature at 450 °C. Ion spray floating was set the voltage at 4500 V in positive mode and at − 4500 V in negative mode. The de-clustering potential was set to 100 V in positive mode and to − 100 V in negative mode. Data were acquired in the informative dependent acquisition mode composed of a TOF–MS scan, and 10 dependent product ion scans were used in the high sensitivity mode with dynamic background subtraction. The collision energy was set to 30 V, and the collision energy spread was set to 15 V. The mass range of the TOF–MS scan was m/z 100–1,000, and the product the ion scan was set to m/z 50–1,000. Equal aliquots of each metabolite sample were pooled to form the quality control (QC) samples. The QC samples were injected before, during, and after sample analysis to assess the system performance.

#### Metabolome analysis and pathway enrichment

The standard format of AB SCIEX proprietary (.wiff and .wiff.scan) were converted into XML-based format (.mzML) by MSConvert from the ProteoWizard^44^. Afterward, the peak alignment and determination of each metabolite were performed by the default parameters of "UPLC/Triple TOF" protocol implemented in XCMS online (version 3.7.1) (The Scripps Research Institute, CA, USA). In brief, the highly sensitive feature detection using a centWave algorithm^45^ was composed of maximal tolerated m/z deviation at 15 ppm, 5–20 s peak width of chromatographic and 0.01 minimum difference in m/z for peaks with overlapping retention times. Subsequently, the total mass spectral of metabolites was identified to individual m/z features by the METLIN database (http://metlin.scripps.edu). Statistical analysis was performed using the univariate (Volcano plot) and multivariate (Partial Least Squares-Discriminant Analysis, PLS-DA) by MetaboAnalyst (version 5.0)^46^. Pathway analysis was performed using the Mummichog algorithm^47^. These prominent pathways were depicted based on the nematode (*Caenorhabditis elegans*) in the Kyoto Encyclopedia of Genes and Genomes (KEGG) pathway database (www.genome.jp/kegg)^48–50^ .

### Integrative and association analysis of bacteriome and metabolome

The summarized data including the abundance of bacterial genus and the most important metabolite features was performed using xMWAS online (v0.552) (https://kuppal.shinyapps.io/xmwas/) to understand the association between gut bacteriome and metabolomic. Briefly, xMWAS constructs a pairwise correlation analysis of data using Partial Least Squares (PLS) methods, then integration and filters the top association scores by Student’s *t*-test (*P* < 0.05) and correlation threshold. Furthermore, centrality scores are compared to generate differences between groups based on eigenvector centrality and the edges lists are utilized to group the community upon which detection algorithms.

### Ethical approval

The study protocol was approved by the ethics committees of the Faculty of Tropical Medicine, Mahidol University (MUTM 2021–020-01). Written informed consent was obtained from each subject before the study.

## Supplementary Information

Supplementary Information 1.

Supplementary Information 2.

Supplementary Information 3.

Supplementary Information 4.

Supplementary Information 5.

Supplementary Information 6.

Supplementary Information 7.

Supplementary Information 8.

Supplementary Information 9.

## Data Availability

The data obtained from this study is available at NCBI database: BioProject ID PRJNA879059. (https://dataview.ncbi.nlm.nih.gov/object/PRJNA879059?reviewer=tdvnt8agbn7k3svksh0njcbn81).
