# Supplementary figures and images for: Gut bacteriome and metabolome of Ascaris lumbricoides in patients
Source: Sci Rep. 2022 Nov 14;12:19524. doi: 10.1038/s41598-022-23608-9 (PMC9663418; doi:10.1038/s41598-022-23608-9)

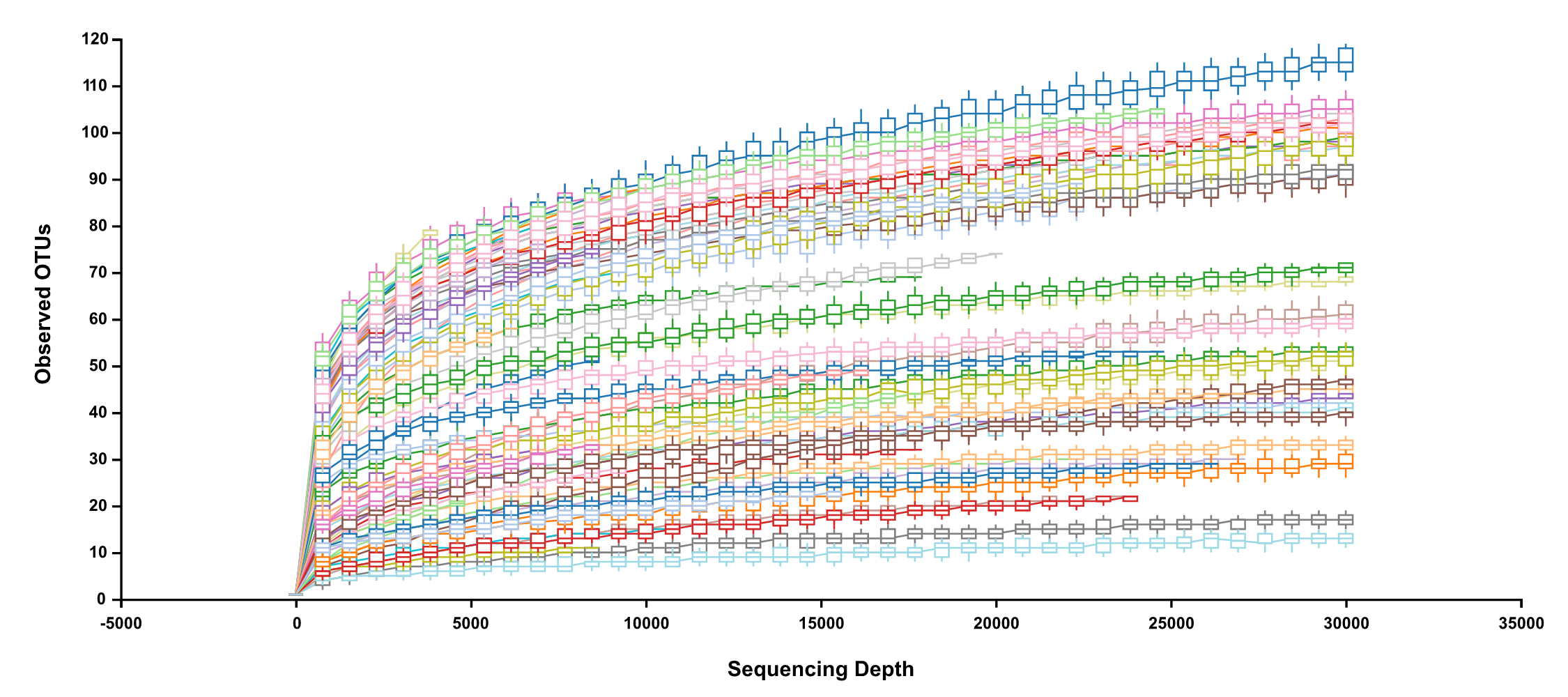

Supplement: Supplementary file 1 — Supplementary Information 1. [file 41598_2022_23608_MOESM1_ESM.tif]

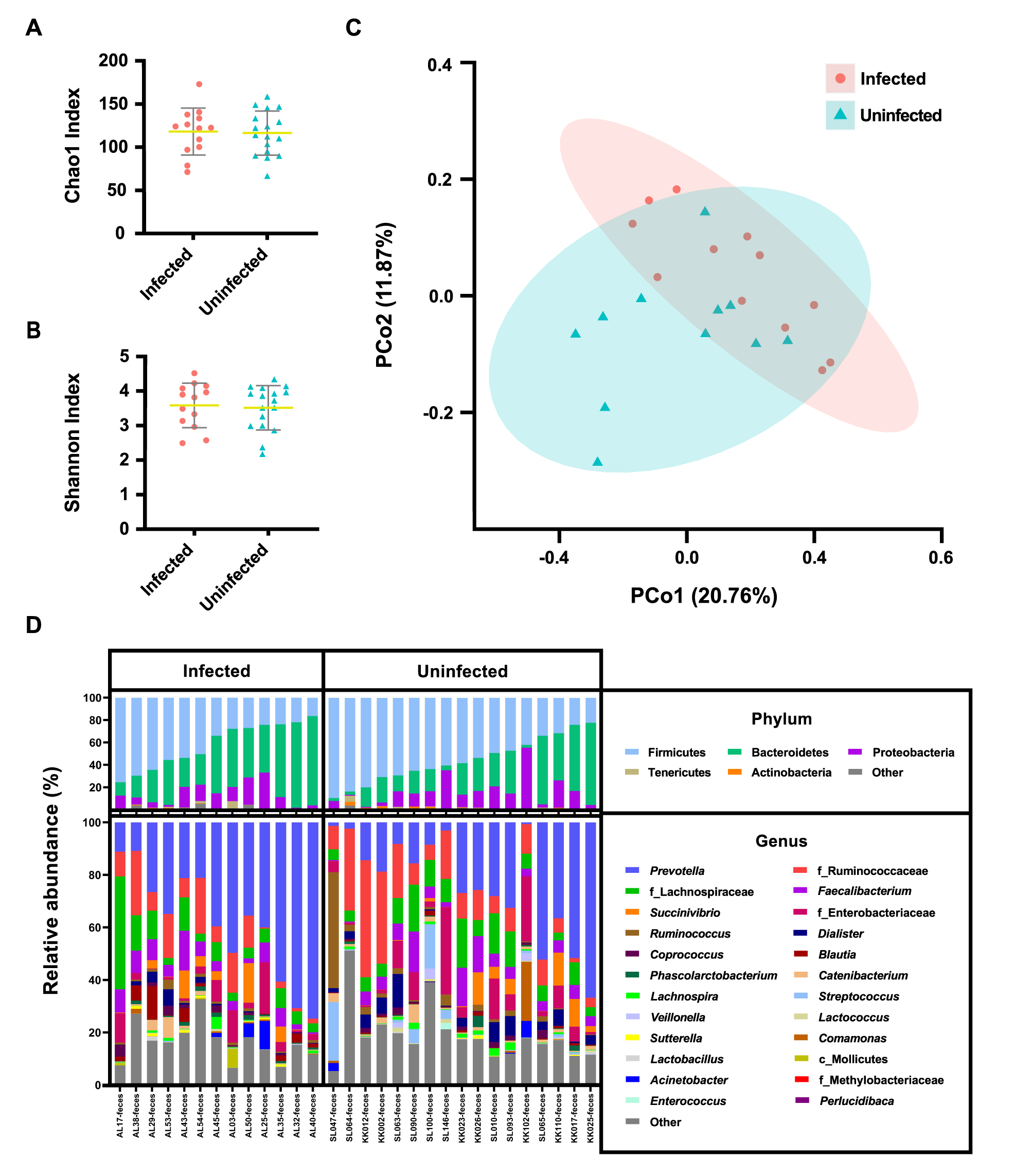

Supplement: Supplementary file 3 — Supplementary Information 3. [file 41598_2022_23608_MOESM3_ESM.tif]

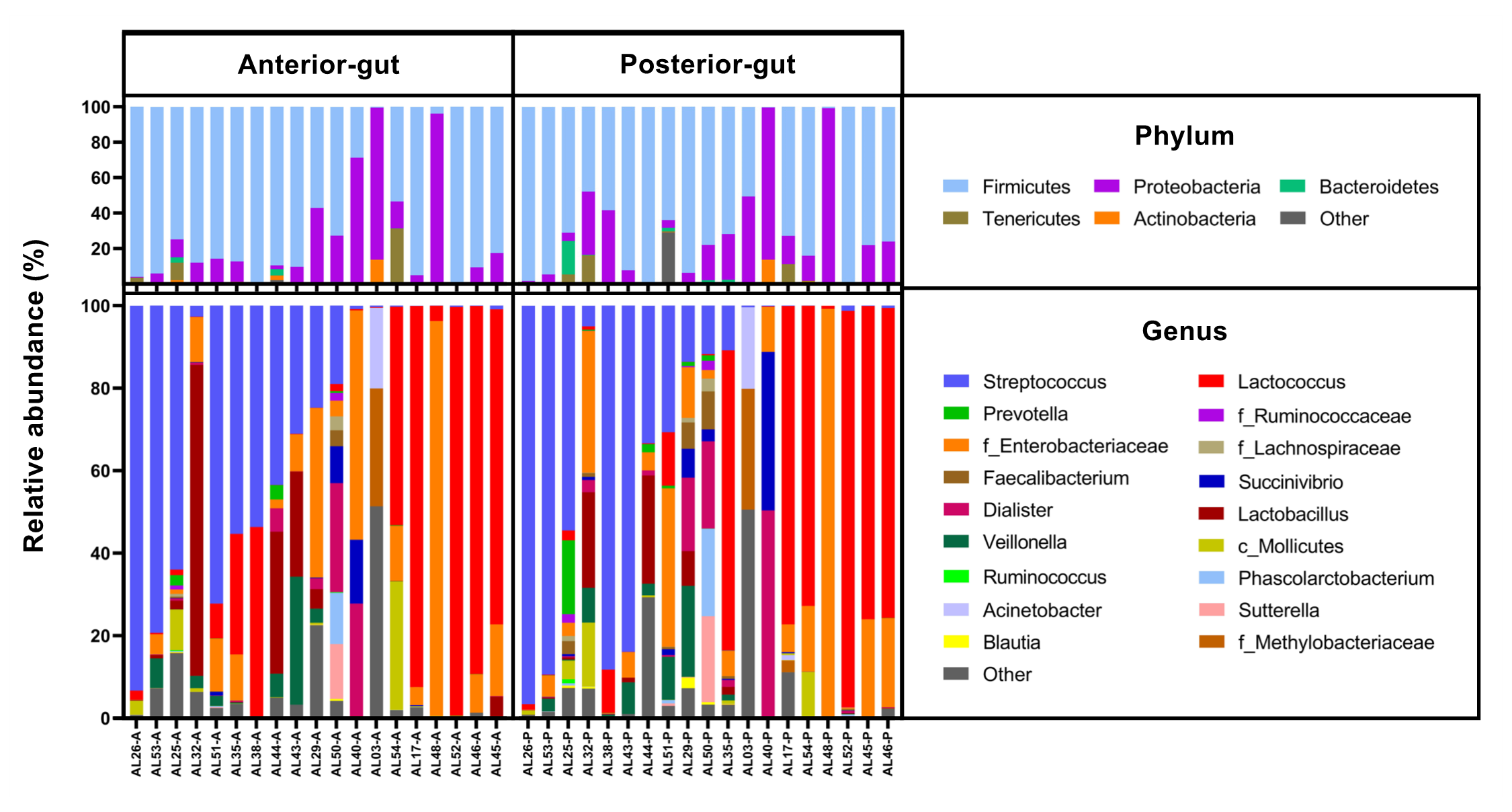

Supplement: Supplementary file 5 — Supplementary Information 5. [file 41598_2022_23608_MOESM5_ESM.tif]

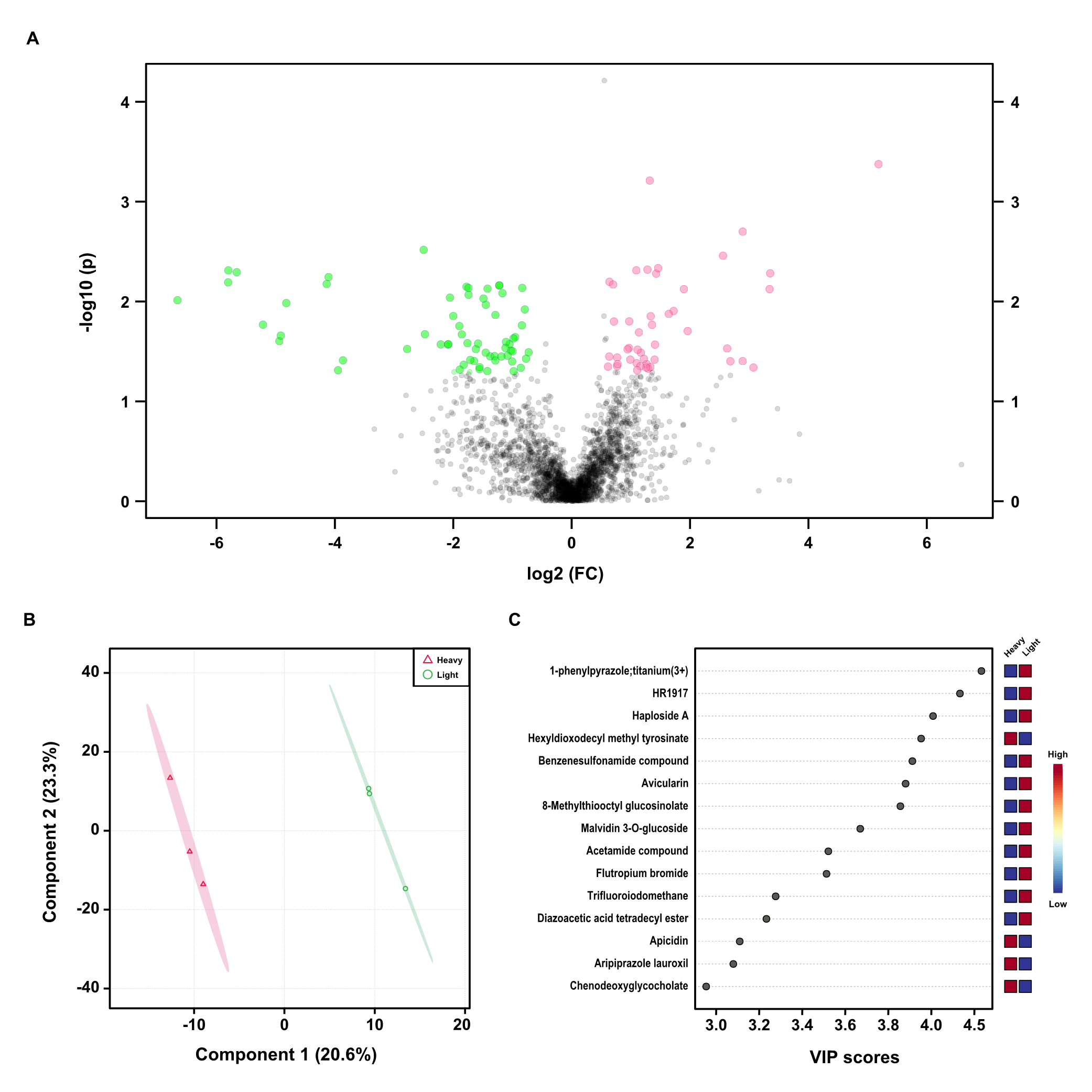

Supplement: Supplementary file 7 — Supplementary Information 7. [file 41598_2022_23608_MOESM7_ESM.tif]
