# Supplementary material for: Gut bacteriome and metabolome of Ascaris lumbricoides in patients
Source: Sci Rep. 2022 Nov 14;12:19524. doi: 10.1038/s41598-022-23608-9 (PMC9663418; doi:10.1038/s41598-022-23608-9)
Supplement: Supplementary file 2 — Supplementary Information 2. [file 41598_2022_23608_MOESM2_ESM.docx]

**Supplementary Table S1. Table of top 30 differentiating metabolites by VIP score in the positive ion and negative ion modes between the heavy and light infected ascariasis.**

| Name | VIP score | Input m/z | Adduct | Actual Mass | Database Match | Formula | Tolerance (ppm) |
| --- | --- | --- | --- | --- | --- | --- | --- |
| M529T6_2 | 4.577 | 529.1207506 | [M+H]+ | 528.1111 | Triphenylstannyl 4-tert-butylbenzoate | C29H28O2Sn | 4 |
| M512T6_3 | 4.2326 | 512.0992644 | [M+Cl]- | 477.1307 | 1-phenylpyrazole;titanium(3+) | C27H21N6Ti | 1 |
| M568T6_2 | 4.1329 | 568.1222454 | [M-H]- | 569.1345 | HR1917 | C26H25Cl2N7O4 | 8 |
| M535T6_2 | 4.0079 | 535.1097868 | [M-H]- | 536.1166 | Haploside A | C24H24O14 | 0 |
| M446T1_17 | 3.9524 | 446.2882777 | [M-H]- | 447.2985 | Hexyldioxodecyl methyl tyrosinate | C26H41NO5 | 6 |
| M527T6_2 | 3.9118 | 527.1076665 | [M+Cl]- | 492.1387 | N-[3-(2-amino-4-chloro-6-phenylpyrimidin-5-yl)propyl]-4-methyl-N-phenylbenzenesulfonamide | C26H25ClN4O2S | 0 |
| M469T6_2 | 3.8803 | 469.0562059 | [M+Cl]- | 434.0849 | Avicularin | C20H18O11 | 4 |
| M512T6_2 | 3.856 | 512.0840082 | [M+Cl]- | 477.1161 | 8-Methylthiooctyl glucosinolate | C16H31NO9S3 | 2 |
| M440T9_13 | 3.8112 | 440.2820361 | [M+H]+ | 439.2732 | Ethanesulfonic acid, 2-(cyclohexyl(1-oxohexadecyl)amino)-, sodium salt | C22H42NNaO4S | 3 |
| M527T6_1 | 3.6695 | 527.0948704 | [M+Cl]- | 492.1268 | Malvidin 3-O-glucoside | C23H24O12 | 2 |
| M313T12_13 | 3.5638 | 313.3197169 | [M+H]+ | 312.3141 | Methyloctadecylnitrosamine | C19H40N2O | 5 |
| M331T12_12 | 3.5425 | 331.326552 | [M+H]+ | 330.3134 | Hexadecylmethylglycerol | C20H42O3 | 17 |
| M527T1_17 | 3.5213 | 527.10794 | [M+Cl]- | 492.1387 | N-(4-chlorophenyl)-2-[[5-[(2,6-dimethylphenoxy)methyl]-4-(4-methylphenyl)-1,2,4-triazol-3-yl]sulfanyl]acetamide | C26H25ClN4O2S | 0 |
| M512T1_12 | 3.5127 | 512.099462 | [M+Cl]- | 477.1315 | Flutropium bromide | C24H29BrFNO3 | 2 |
| M195T1_4 | 3.2763 | 194.8902899 | [M-H]- | 195.8997 | Trifluoroiodomethane | CF3I | 10 |
| M281T13_6 | 3.2334 | 281.224263 | [M-H]- | 282.2307 | Diazoacetic acid tetradecyl ester | C16H30N2O2 | 2 |
| M341T13_27 | 3.1872 | 341.3494984 | [M+H]+ | 340.3454 | 1,1,3,3-tetrapentylurea | C21H44N2O | 9 |
| M338T14_15 | 3.1332 | 338.3130258 | [M+Na]+ | 315.3217 | Arachidic acid(d3) | C20H37D3O2 | 6 |
| M658T1_12 | 3.1094 | 658.3349903 | [M+Cl]- | 623.3683 | Apicidin | C34H49N5O6 | 4 |
| M658T1_11 | 3.0796 | 658.3213897 | [M-H]- | 659.3257 | Aripiprazole lauroxil | C36H51Cl2N3O4 | 4 |
| M759T0_8 | 3.0694 | 758.6330519 | [M+Na]+ | 735.6563 | Benzenesulfonate;tetrakis-decylazanium | C46H89NO3S | 16 |
| M467T10_5 | 3.0689 | 467.3040459 | [M+H]+ | 467.3040459 | N-Methyl-N-octadecyl-3-nitro-4-chlorobenzamide | C26H43ClN2O3 | 1 |
| M175T1_11 | 3.0562 | 175.0700452 | [M+H]+ | 174.0632 | 2-chloroethynyl(triethyl)silane | C8H15ClSi | 2 |
| M677T14_14 | 2.9555 | 676.7465509 | [M+Na]+ | 653.7615 | 11-(Pentabromophenoxy)undecan-1-ol | C17H23Br5O2 | 6 |
| M448T1_14 | 2.9534 | 448.3039998 | [M-H]- | 449.3141 | Chenodeoxyglycocholate | C26H43NO5 | 6 |
| M483T10_19 | 2.9159 | 483.4248603 | [M+H]+ | 482.4124 | N-[2-(1H-Indol-1-yl)ethyl]docosanamide | C33H54O2 | 10 |
| M774T15_9 | 2.8322 | 773.6195384 | [M+Na]+ | 750.6315 | Plastoquinol-9 | C53H82O2 | 1 |
| M730T16_7 | 2.8132 | 729.5714123 | [M+H]+ | 728.56 | Trifluoromethanesulfonic acid 2,3-bis(octadecyloxy)propyl ester | C40H79F3O5S | 5 |
| M340T14_36 | 2.8061 | 340.3158195 | [M+H]+ | 339.3137 | Methyl oleoylethanolamide | C21H41NO2 | 15 |
| M445T12_6 | 2.8055 | 445.3820805 | [M+Na]+ | 422.3889 | Phosphine oxide, trioctyl-, dihydrate | C24H55O3P | 8 |
