# Supplementary material for: Gut bacteriome and metabolome of Ascaris lumbricoides in patients
Source: Sci Rep. 2022 Nov 14;12:19524. doi: 10.1038/s41598-022-23608-9 (PMC9663418; doi:10.1038/s41598-022-23608-9)
Supplement: Supplementary file 4 — Supplementary Information 4. [file 41598_2022_23608_MOESM4_ESM.docx]

**S2 Table. The order of KEGG enriched pathways was displayed based on total compounds and *P*-value.**

| Pathways | Total compounds | *P*-value | Enrichment factor |
| --- | --- | --- | --- |
| Amino sugar and nucleotide sugar metabolism* | 20 | 0.006516 | 2.863688 |
| Fructose and mannose metabolism* | 12 | 0.009559 | 3.409013 |
| Polyketide sugar unit biosynthesis* | 2 | 0.014748 | 8.181967 |
| Glycosylphosphatidylinositol (GPI)-anchor biosynthesis* | 12 | 0.047509 | 2.727211 |
| Glycosaminoglycan degradation | 25 | 0.07222 | 1.963608 |
| Glycosaminoglycan biosynthesis - heparan sulfate / heparin | 4 | 0.075003 | 4.0909 |
| Lysine degradation | 14 | 0.079413 | 2.337678 |
| Pentose and glucuronate interconversions | 10 | 0.11171 | 2.45459 |
| Galactose metabolism | 11 | 0.14052 | 2.231479 |
| Steroid biosynthesis | 26 | 0.20134 | 1.573416 |
| Arachidonic acid metabolism | 26 | 0.20134 | 1.573416 |
| Starch and sucrose metabolism | 7 | 0.2062 | 2.33765 |
| Glycosaminoglycan biosynthesis - chondroitin sulfate / dermatan sulfate | 7 | 0.2062 | 2.33765 |
| Pentose phosphate pathway | 27 | 0.22463 | 1.515152 |
| Glycosphingolipid biosynthesis - lacto and neolacto series | 2 | 0.22936 | 4.090983 |
| Glycosphingolipid biosynthesis - globo and isoglobo series | 2 | 0.22936 | 4.090983 |
| Glycerolipid metabolism | 8 | 0.25413 | 2.04545 |
| Porphyrin and chlorophyll metabolism | 23 | 0.30521 | 1.422931 |
| Phosphatidylinositol signaling system | 10 | 0.34995 | 1.636393 |
| Purine metabolism | 65 | 0.3959 | 1.132873 |
| Phosphonate and phosphinate metabolism | 4 | 0.40657 | 2.04545 |
| Glycolysis or Gluconeogenesis | 12 | 0.44149 | 1.363605 |
| Inositol phosphate metabolism | 12 | 0.44149 | 1.363605 |
| alpha-Linolenic acid metabolism | 5 | 0.4794 | 1.636367 |
| Sulfur metabolism | 5 | 0.4794 | 1.636367 |
| Ascorbate and aldarate metabolism | 6 | 0.54339 | 1.363643 |
| Caffeine metabolism | 6 | 0.54339 | 1.363643 |
| Phenylalanine, tyrosine and tryptophan biosynthesis | 6 | 0.54339 | 1.363643 |
| Drug metabolism - cytochrome P450 | 16 | 0.60103 | 1.022704 |
| Terpenoid backbone biosynthesis | 18 | 0.66716 | 0.9090909 |
| Arginine and proline metabolism | 28 | 0.68646 | 0.8766291 |
| Phenylalanine metabolism | 9 | 0.69227 | 0.9090909 |
| Sphingolipid metabolism | 29 | 0.71013 | 0.8464056 |
| N-Glycan biosynthesis | 21 | 0.74966 | 0.7792107 |
| Glycerophospholipid metabolism | 22 | 0.77302 | 0.7437986 |
| Mannose type O-glycan biosynthesis | 12 | 0.79297 | 0.6818027 |
| Drug metabolism - other enzymes | 23 | 0.79448 | 0.7114653 |
| One carbon pool by folate | 13 | 0.81867 | 0.6293662 |
| Aminoacyl-tRNA biosynthesis | 35 | 0.82447 | 0.7012951 |
| Folate biosynthesis | 25 | 0.83216 | 0.6545359 |
| Fatty acid degradation | 14 | 0.84121 | 0.5844194 |
| Nicotinate and nicotinamide metabolism | 14 | 0.84121 | 0.5844194 |
| Pyrimidine metabolism | 47 | 0.85032 | 0.6963303 |
| Glyoxylate and dicarboxylate metabolism | 15 | 0.86097 | 0.5454645 |
| Metabolism of xenobiotics by cytochrome P450 | 17 | 0.89349 | 0.4812783 |
| Alanine, aspartate and glutamate metabolism | 18 | 0.9068 | 0.4545455 |
| Retinol metabolism | 19 | 0.91847 | 0.4306261 |
| Cysteine and methionine metabolism | 33 | 0.92845 | 0.4958719 |
| Tryptophan metabolism | 20 | 0.92869 | 0.4090983 |
| Pantothenate and CoA biosynthesis | 22 | 0.94548 | 0.3718993 |
| Glycine, serine and threonine metabolism | 23 | 0.95235 | 0.3557326 |
