# Supplementary material for: Gut bacteriome and metabolome of Ascaris lumbricoides in patients
Source: Sci Rep. 2022 Nov 14;12:19524. doi: 10.1038/s41598-022-23608-9 (PMC9663418; doi:10.1038/s41598-022-23608-9)
Supplement: Supplementary file 6 — Supplementary Information 6. [file 41598_2022_23608_MOESM6_ESM.docx]

**S3 Table. Clustered of integrative networks between relative abundance of bacteria and metabolites in the positive and negative modes.**

| Community | Bacteria | Node | Code Name (Ion modes) | Input m/z | Database Match |
| --- | --- | --- | --- | --- | --- |
| 1 | *Prevotella, Faecalibacterium, Succinivibrio*, Ruminococcaceae and Lachnospiraceae | Y125 | M299T9_13 (N) | 299.2885 | 2-Octylundecane-1,11-diol |
|  |  | Y149 | M694T10_8 (N) | 694.3559 | Jubanine A |
|  |  | Y156 | M269T1_11 (N) | 268.9833 | Diethyl (2R,3R)-2-bromo-3-fluorobutanedioate |
|  |  | Y162 | M340T10_1 (N) | 340.1761 | Acetylintermedine |
|  |  | Y168 | M674T6_2 (N) | 674.3194 | Jesaconitine |
|  |  | Y170 | M628T10_12 (N) | 628.386 | (4R)-4-Benzyl-3-{(2R,3S,4R,5S,6S)-3-{[tert-butyl(dimethyl)silyl]oxy}-2,4,6-trimethyl-5-[(triethylsilyl)oxy]deca-7,9-dienoyl}-1,3-oxazolidin-2-one |
|  |  | Y21 | M408T12_3 (P) | 408.3089 | Cassaidine |
|  |  | Y59 | M628T10_5 (P) | 628.379 | Pro Glu Arg Val Lys |
|  |  | Y60 | M468T11_39 (P) | 468.3641 | Sulfuric acid, monooctadecyl ester, compd. with 2-(diethylamino)ethanol (1:1) |
|  |  | Y77 | M628T10_6 (P) | 628.3952 | 5-{[3-(Octadecyloxy)propyl]carbamoyl}benzene-1,2,3-triyl triacetate |
| 2 | Enterobacteriaceae | Y131 | M283T15_1 (N) | 283.2637 | 2-hexyldodecanoic acid |
|  |  | Y136 | M596T9_12 (N) | 596.357 | Molybdenum, compd. with nickel (1:8) |
|  |  | Y165 | M526T9_3 (N) | 526.2584 | Glutathionylspermine |
|  |  | Y28 | M291T1_10 (P) | 291.1149 | Butylphosphonic acid diphenyl ester |
|  |  | Y38 | M438T9_10 (P) | 438.337 | (+/-)N-(1-methyl-2-hydroxy-2-phenyl-ethyl) arachidonyl amine |
|  |  | Y4 | M331T12_12 (P) | 331.3266 | Hexadecylmethylglycerol |
|  |  | Y67 | M287T1_6 (P) | 287.0132 | 3,6-Di(ethanesulfonyl)pyridazine |
|  |  | Y75 | M482T10_27 (P) | 482.422 | N-Methyl-N,N-dioctyloctan-1-aminium trifluoroacetate |
|  |  | Y95 | M469T6_2 (N) | 469.0562 | avicularin |
| 3 | *Streptococcus* and *Lactococcus* | Y133 | M387T1_12 (N) | 387.0097 | Ethyl 7-(2,4,5-trichlorophenoxy)heptanoate |
|  |  | Y147 | M221T7_2 (N) | 221.1011 | Thiobenzoic acid S-hexyl ester |
|  |  | Y148 | M640T9_10 (N) | 640.3491 | Leu Leu Val Val Tyr |
|  |  | Y172 | M616T13_5 (N) | 615.5433 | Dioctadecyl thiourea |
|  |  | Y178 | M832T14_4 (N) | 831.5859 | 16:2-Glc-Campesterol |
|  |  | Y182 | M409T10_2 (N) | 409.2349 | LPA(0:0/16:0) |
|  |  | Y36 | M670T10_7 (P) | 670.3949 | L-Phenylalanylglycyl-L-leucyl-L-alanyl-L-lysyl-L-leucine |
|  |  | Y62 | M487T11_9 (P) | 487.2923 | Gln Ile Gln Val |
|  |  | Y124 | M628T10_10 (N) | 628.3551 | Arg Val Ile Trp Gly |
|  |  | Y127 | M803T0_6 (N) | 802.6196 | PE(16:0/24:0) |
|  |  | Y150 | M382T13_10 (N) | 382.2187 | 6,7-Dihydro-8-phenyl-9-(4-(2-(dimethylamino)ethoxy)phenyl)-5-H-benzocycloheptene |
|  |  | Y159 | M441T10_3 (N) | 441.2532 | N-linolenoyl-glutamine |
|  |  | Y174 | M236T0_1 (N) | 235.9143 | Selenophene-2-carbonyl azide |
|  |  | Y177 | M507T10_5 (N) | 507.3006 | Reticulataxanthinz |
|  |  | Y112 | M300T1_6 (N) | 300.0495 | N-Acetyl-D-galactosamine 6-phosphate |
|  |  | Y142 | M630T10_8 (N) | 630.3741 | Jurubine |
|  |  | Y153 | M480T9_3 (N) | 480.2776 | Urea, N-(2,6-bis(1-methylethyl)phenyl)-N'-((1-(1,3-dimethyl-1H-indol-2-yl)cyclopentyl)methyl)- |
|  |  | Y17 | M783T17_4 (P) | 782.5792 | PC(20:3(8Z,11Z,14Z)/16:1(9Z)) |
|  |  | Y18 | M359T13_11 (P) | 359.2989 | Propanoic acid, 3-mercapto-, octadecyl ester |
|  |  | Y181 | M830T0_8 (N) | 829.6348 | 2,3,4,5-Tetraiodo-6-[(naphthalen-1-yl)carbamoyl]benzoic acid |
|  |  | Y100 | M195T1_4 (N) | 194.8903 | Trifluoroiodomethane |
|  |  | Y103 | M658T1_11 (N) | 658.3214 | Aripiprazole lauroxil |
|  |  | Y114 | M658T6_4 (N) | 658.3427 | Arg Arg Arg His |
|  |  | Y12 | M774T15_9 (P) | 773.6195 | Plastoquinol-9 |
|  |  | Y16 | M345T14_12 (P) | 345.2968 | 2-Phenyl-4-tetradecyl-1,3,2-dioxaborolane |
|  |  | Y33 | M339T12_9 (P) | 339.3213 | 13(Z)-Docosenoic Acid |
|  |  | Y8 | M467T10_5 (P) | 467.304 | N-Methyl-N-octadecyl-3-nitro-4-chlorobenzamide |
|  |  | Y93 | M446T1_17 (N) | 446.2883 | Hexyldioxodecyl methyl tyrosinate |
|  |  | Y90 | M512T6_3 (N) | 512.0993 | 1-phenylpyrazole;titanium(3+) |
|  |  | Y91 | M568T6_2 (N) | 568.1222 | 4',4''-Di-2-imidazolin-2-yl-2-nitroterephthalanilide dihydrochloride |
|  |  | Y92 | M535T6_2 (N) | 535.1098 | Haploside A |
|  |  | Y7 | M759T0_8 (P) | 758.6331 | Benzenesulfonate;tetrakis-decylazanium |
|  |  | Y69 | M522T9_21 (P) | 522.3917 | 3-[3,4-Bis(decyloxy)phenyl]-2-(ethoxymethyl)prop-2-enenitrile |
|  |  | Y61 | M487T11_6 (P) | 487.2788 | Pristimerin |
|  |  | Y39 | M265T1_6 (P) | 265.0237 | Azelaic acid, potassium salt |
|  |  | Y27 | M603T11_4 (P) | 603.4063 | (3beta)-3-{[(2Z)-3-(4-Hydroxyphenyl)prop-2-enoyl]oxy}urs-12-en-28-oic acid |
|  |  | Y2 | M440T9_13 (P) | 440.282 | Ethanesulfonic acid, 2-(cyclohexyl(1-oxohexadecyl)amino)-, sodium salt |
|  |  | Y19 | M469T11_35 (P) | 469.3929 | Lupeol acetate |
|  |  | Y140 | M339T10_10 (N) | 339.2299 | 2,4-Bis(4-methylpiperazin-1-yl)-3H-1,5-benzodiazepine |
|  |  | Y130 | M668T10_9 (N) | 668.3775 | 3-(4-Methylpyridin-1-ium-1-yl)cholest-5-ene 4-methylbenzene-1-sulfonate |
|  |  | Y11 | M483T10_19 (P) | 483.4249 | N-[2-(1H-Indol-1-yl)ethyl]docosanamide |
|  |  | Y107 | M568T1_8 (N) | 568.1172 | Idarubicin hydrochloride |
|  |  | Y106 | M527T1_15 (N) | 527.0965 | Malvidin 3-O-glucoside |
|  |  | Y1 | M529T6_2 (P) | 529.1208 | Triphenylstannyl 4-tert-butylbenzoate |
|  |  | Y96 | M512T6_2 (N) | 512.084 | 8-Methylthiooctyl glucosinolate |
|  |  | Y98 | M527T1_17 (N) | 527.1079 | N-(4-chlorophenyl)-2-[[5-[(2,6-dimethylphenoxy)methyl]-4-(4-methylphenyl)-1,2,4-triazol-3-yl]sulfanyl]acetamide |
|  |  | Y99 | M512T1_12 (N) | 512.0995 | Flutropium bromide |
| 4 | *Dialister* | Y155 | M307T13_6 (N) | 307.3084 | Glyodin |
|  |  | Y37 | M631T10_8 (P) | 631.4385 | Dexamethasone palmitate |
|  |  | Y88 | M225T1_9 (P) | 225.0483 | Penmacric acid |
| 5 | *Lactobacillus* | Y14 | M340T14_36 (P) | 340.3158 | (4e)-4-(hexadecylimino)pentanoic acid |
|  |  | Y42 | M630T10_4 (P) | 630.3835 | Trp Leu Arg Arg |
|  |  | Y49 | M319T9_1 (P) | 319.2234 | 3-Oxooctadec-4-enoic acid |
|  |  | Y76 | M338T14_12 (P) | 338.2501 | Suloctidil |
|  |  | Y79 | M602T9_9 (P) | 602.3488 | Asp Arg Arg Arg |
|  |  | Y83 | M598T9_6 (P) | 598.3732 | Lysophosphatidylcholine (22:2) |
