# Supplementary material for: Gut bacteriome and metabolome of Ascaris lumbricoides in patients
Source: Sci Rep. 2022 Nov 14;12:19524. doi: 10.1038/s41598-022-23608-9 (PMC9663418; doi:10.1038/s41598-022-23608-9)
Supplement: Supplementary file 8 — Supplementary Information 8. [file 41598_2022_23608_MOESM8_ESM.docx]

**Supplementary Table S4. Table of the characteristics of gut ascariasis patient (n = 19) in Ban Mae Salid Luang (BL) and Gre Key (GK) village, Thailand.**

| Sample code | Age of human hosts | Sex of human hosts | Infection intensity | Collection date | Geographic location |
| --- | --- | --- | --- | --- | --- |
| AL03 | 3 | Male | Moderate | 06/22/2019 | Thailand: BLvillage |
| AL17 | 8 | Male | Moderate | 06/24/2019 | Thailand: BLvillage |
| AL25 | 9 | Male | Heavy | 06/26/2019 | Thailand: BLvillage |
| AL26 | 9 | Male | Heavy | 06/28/2019 | Thailand: BLvillage |
| AL29 | 13 | Female | Moderate | 06/30/2019 | Thailand: BLvillage |
| AL32 | 11 | Male | Heavy | 07/02/2019 | Thailand: BLvillage |
| AL35 | 9 | Male | Moderate | 07/04/2019 | Thailand: BLvillage |
| AL38 | 8 | Male | Moderate | 07/06/2019 | Thailand: BLvillage |
| AL40 | 9 | Male | Moderate | 07/08/2019 | Thailand: BLvillage |
| AL43 | 8 | Male | Moderate | 07/10/2019 | Thailand: BLvillage |
| AL44 | 8 | Male | Moderate | 07/12/2019 | Thailand: BLvillage |
| AL45 | 4 | Female | Light | 07/14/2019 | Thailand: GKvillage |
| AL46 | 4 | Female | Light | 07/16/2019 | Thailand: GKvillage |
| AL48 | 22 | Female | Moderate | 07/18/2019 | Thailand: GKvillage |
| AL50 | 24 | Female | Moderate | 07/20/2019 | Thailand: GKvillage |
| AL51 | 24 | Female | Moderate | 07/22/2019 | Thailand: GKvillage |
| AL52 | 8 | Female | Light | 07/24/2019 | Thailand: GKvillage |
| AL53 | 9 | Female | Heavy | 07/26/2019 | Thailand: GKvillage |
| AL54 | 22 | Female | Moderate | 07/28/2019 | Thailand: GKvillage |
