# Supplementary material for: Gut bacteriome and metabolome of Ascaris lumbricoides in patients
Source: Sci Rep. 2022 Nov 14;12:19524. doi: 10.1038/s41598-022-23608-9 (PMC9663418; doi:10.1038/s41598-022-23608-9)
Supplement: Supplementary file 9 — Supplementary Information 9. [file 41598_2022_23608_MOESM9_ESM.docx]

Supplementary Information

Gut bacteriome and metabolome of *Ascaris lumbricoides* in patients

Pavit Klomkliew^1,2^, Vorthon Sawaswong^1,2^, Prangwalai Chanchaem^2^, Pattaraporn Nimsamer^2^, Poom Adisakwattana^3^, Orawan Phuphisut^3^, Phornpimon Tipthara^4^, Joel Tarning^4,5^, Sunchai Payungporn^2,6,*^, and Onrapak Reamtong^7,*^

^1^Program in Bioinformatics and Computational Biology, Graduate School, Chulalongkorn University, Bangkok, 10330, Thailand

^2^Research Unit of Systems Microbiology, Faculty of Medicine, Chulalongkorn University, Bangkok 10330, Thailand

^3^Department of Helminthology, Faculty of Tropical Medicine, Mahidol University, Bangkok, Thailand

^4^Mahidol Oxford Tropical Medicine Research Unit, Faculty of Tropical Medicine, Mahidol University, Bangkok, Thailand

^5^Centre for Tropical Medicine and Global Health, Nuffield Department of Clinical Medicine, University of Oxford, Oxford, United Kingdom

^6^Department of Biochemistry, Faculty of Medicine, Chulalongkorn University, Bangkok 10330, Thailand

^7^Department of Molecular Tropical Medicine and Genetics, Faculty of Tropical Medicine, Mahidol University, Bangkok, 10400 Thailand

^*^Corresponding authors: [onrapak.rea@mahidol.ac.th](mailto:onrapak.rea@mahidol.ac.th) (O.R.); [sp.medbiochemcu@gmail.com](mailto:sp.medbiochemcu@gmail.com) (S.P.)

**Supplementary Figures**


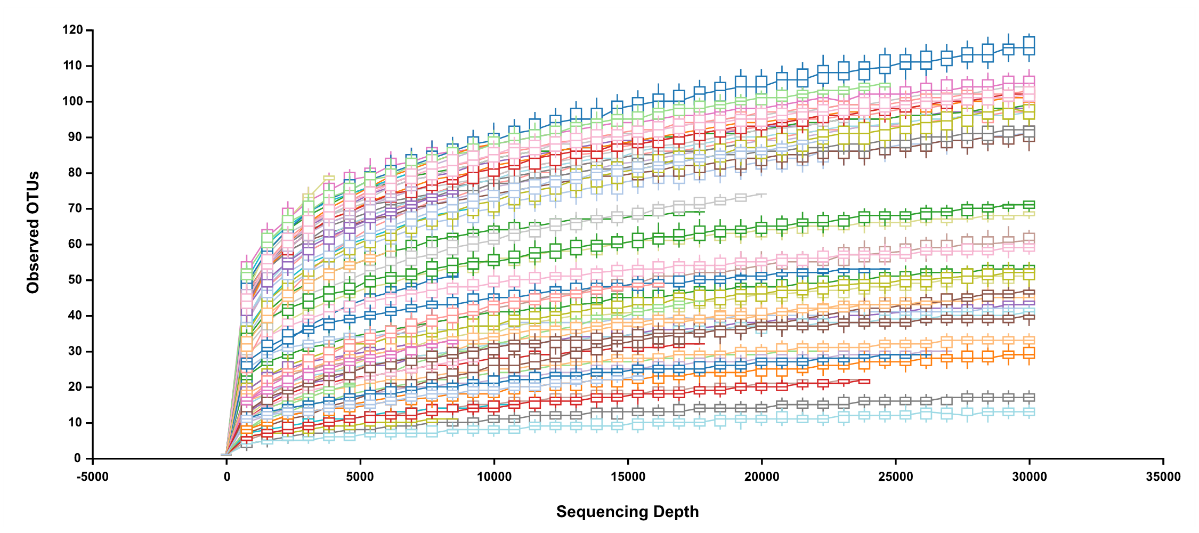


**Supplementary Figures S1.** The rarefaction curve analysis was plotted cumulatively for all groups.


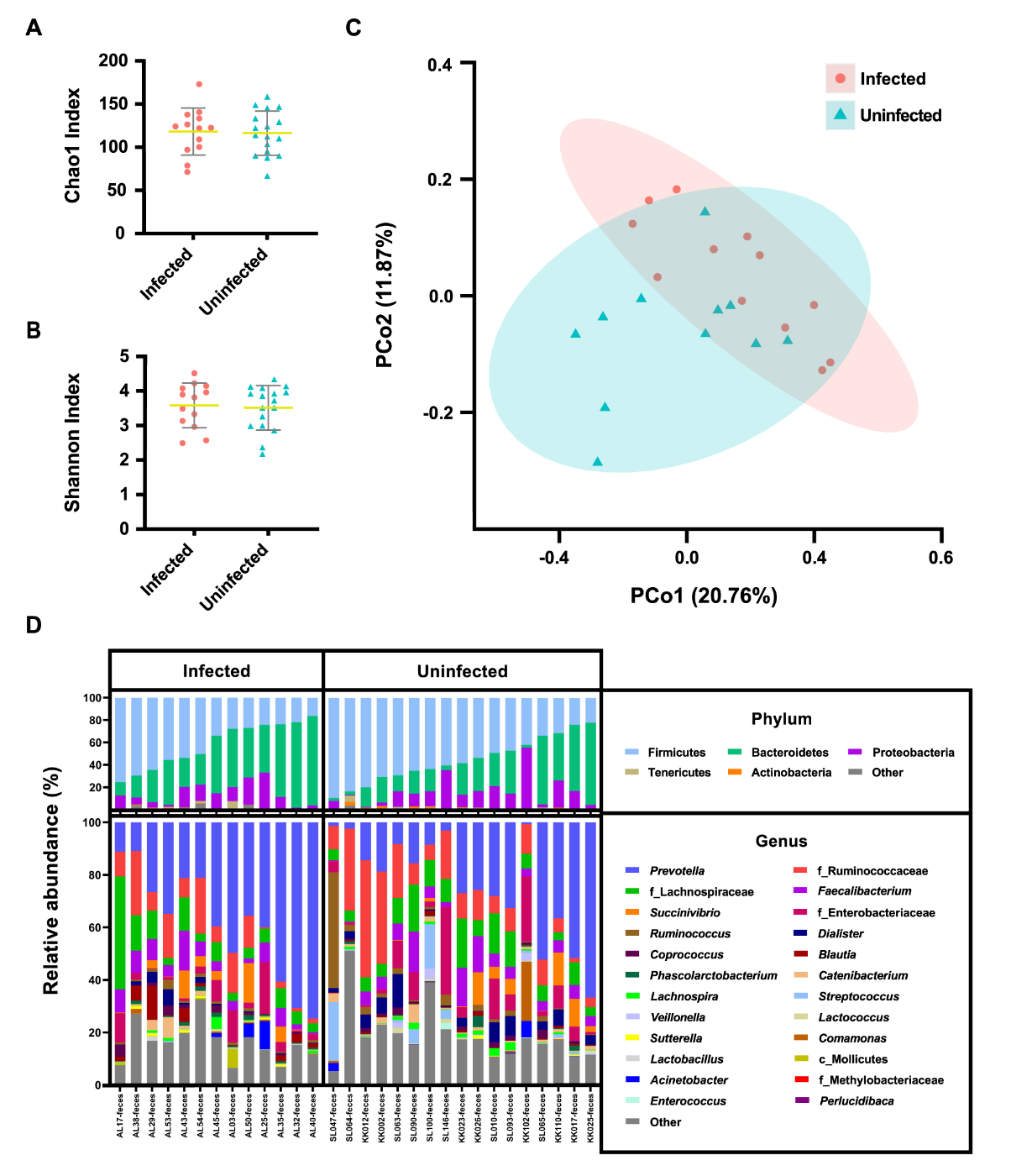


**Supplementary Figures S2.** Gut bacteriome diversity and composition in humans. The alpha diversity comparison of bacteriome in the ascariasis patients and uninfected human hosts calculated by (A) Chao1 index and (B) Shannon indexes were shown as scatter plots with the error bars representing the standard deviation and performed statistically significant difference by Mann-Whitney U test (P < 0.05). (C) The beta diversity of bacteriome between ascariasis patients and uninfected human hosts was presented by principal coordinate analysis (PCoA) plots based on Bray-Curtis distance. (D) The stacked plot showed relative abundances of gut bacteriome at the phylum and genus levels in the feces of ascariasis patients and uninfected human hosts assessed by high throughput sequencing on the 16S rRNA gene.


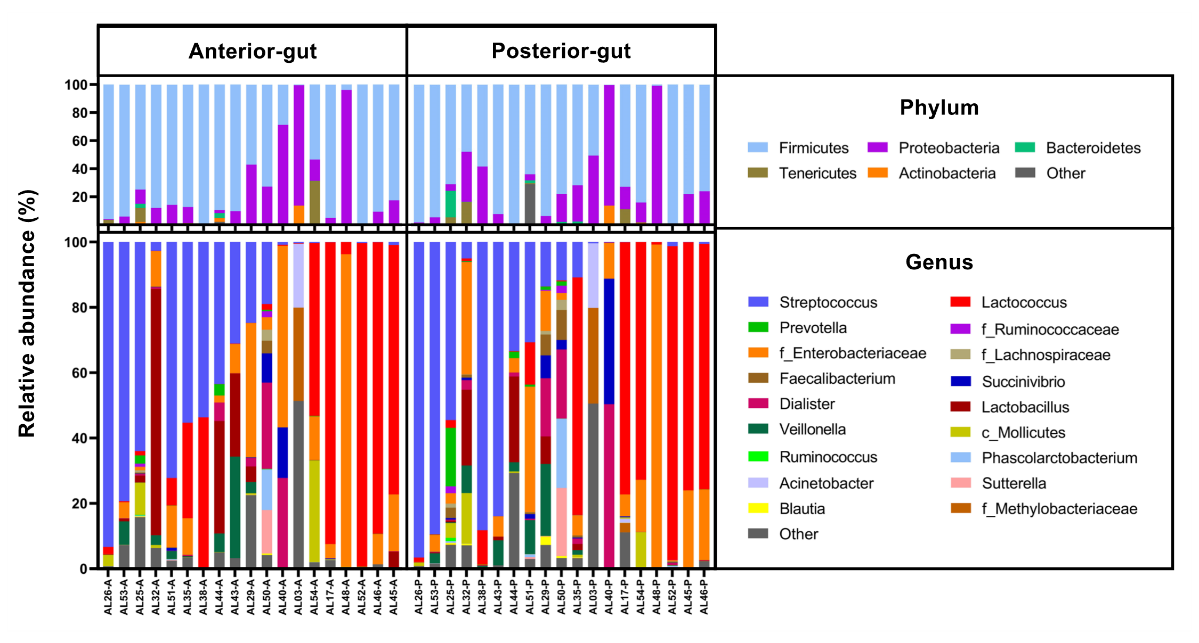
**Supplementary Figures S3.** The relative abundances of gut bacteriome at the phylum and genus levels in the anterior and posterior gut of large roundworms were assessed by high throughput sequencing on the 16S rRNA gene.
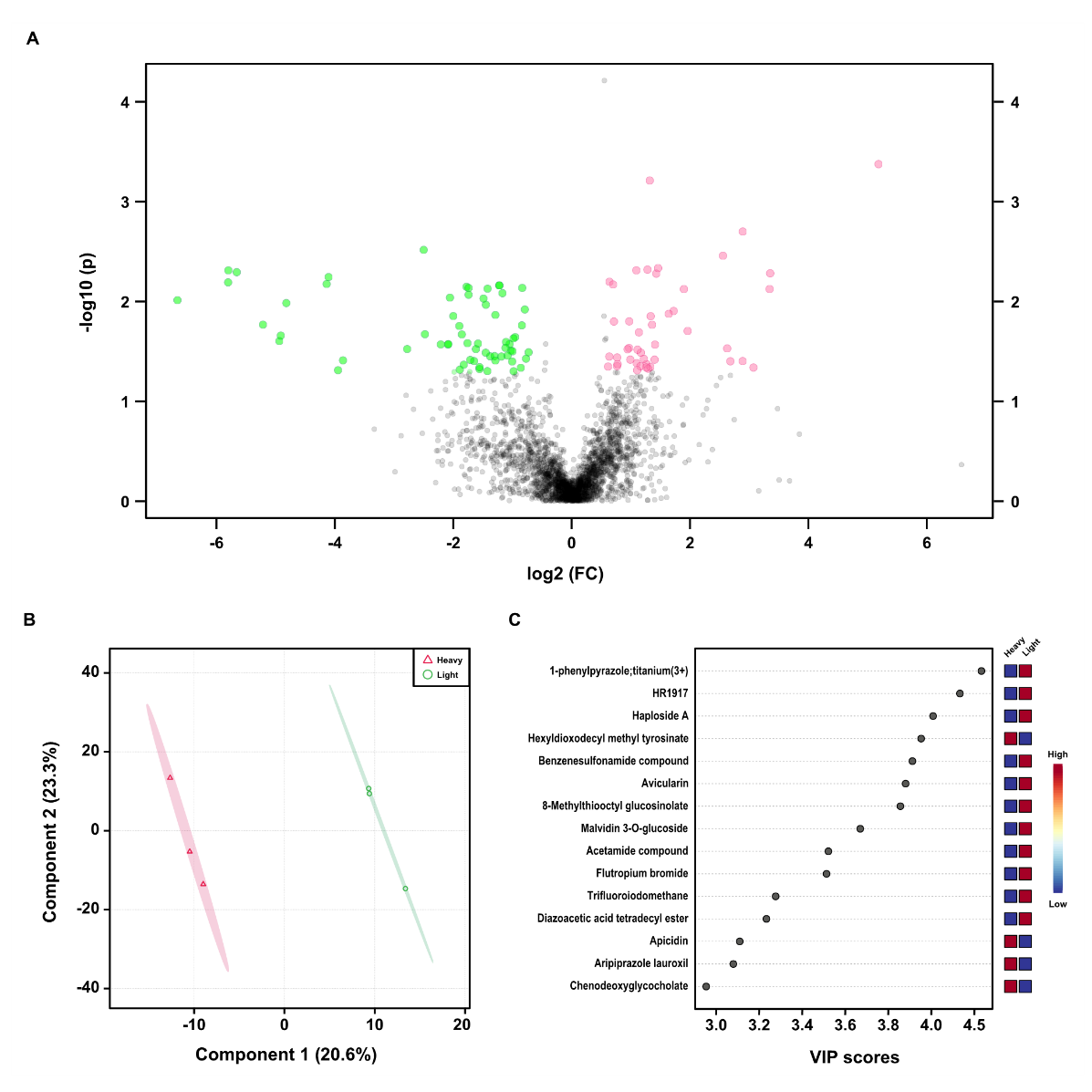


**Supplementary Figures S4.** Gut metabolomics for quantification of metabolites based on negative ion mode between the heavy and light infected ascariasis. (A) Volcano plot showing the statistical significance and fold change of metabolites in heavy infected (red) compared to light infected (green) of ascariasis (*P* < 0.05, Fold change ≥1.5). (B) Score plots of partial least squares discriminant (PLS-DA) analysis of metabolome from heavy and light infected samples. The color around each group represents the 95% confidence intervals. (C) The plot representing 15 m/z features with top variable importance in projection (VIP) score calculated based on PLS-DA analysis.

**Supplementary Tables**

**Supplementary Table S1.** Table of top 30 differentiating metabolites by VIP score in the positive ion and negative ion modes between the heavy and light infected ascariasis.

| Name | VIP score | Input m/z | Adduct | Actual Mass | Database Match | Formula | Tolerance (ppm) |
| --- | --- | --- | --- | --- | --- | --- | --- |
| M529T6_2 | 4.577 | 529.1207506 | [M+H]+ | 528.1111 | Triphenylstannyl 4-tert-butylbenzoate | C29H28O2Sn | 4 |
| M512T6_3 | 4.2326 | 512.0992644 | [M+Cl]- | 477.1307 | 1-phenylpyrazole;titanium(3+) | C27H21N6Ti | 1 |
| M568T6_2 | 4.1329 | 568.1222454 | [M-H]- | 569.1345 | HR1917 | C26H25Cl2N7O4 | 8 |
| M535T6_2 | 4.0079 | 535.1097868 | [M-H]- | 536.1166 | Haploside A | C24H24O14 | 0 |
| M446T1_17 | 3.9524 | 446.2882777 | [M-H]- | 447.2985 | Hexyldioxodecyl methyl tyrosinate | C26H41NO5 | 6 |
| M527T6_2 | 3.9118 | 527.1076665 | [M+Cl]- | 492.1387 | N-[3-(2-amino-4-chloro-6-phenylpyrimidin-5-yl)propyl]-4-methyl-N-phenylbenzenesulfonamide | C26H25ClN4O2S | 0 |
| M469T6_2 | 3.8803 | 469.0562059 | [M+Cl]- | 434.0849 | Avicularin | C20H18O11 | 4 |
| M512T6_2 | 3.856 | 512.0840082 | [M+Cl]- | 477.1161 | 8-Methylthiooctyl glucosinolate | C16H31NO9S3 | 2 |
| M440T9_13 | 3.8112 | 440.2820361 | [M+H]+ | 439.2732 | Ethanesulfonic acid, 2-(cyclohexyl(1-oxohexadecyl)amino)-, sodium salt | C22H42NNaO4S | 3 |
| M527T6_1 | 3.6695 | 527.0948704 | [M+Cl]- | 492.1268 | Malvidin 3-O-glucoside | C23H24O12 | 2 |
| M313T12_13 | 3.5638 | 313.3197169 | [M+H]+ | 312.3141 | Methyloctadecylnitrosamine | C19H40N2O | 5 |
| M331T12_12 | 3.5425 | 331.326552 | [M+H]+ | 330.3134 | Hexadecylmethylglycerol | C20H42O3 | 17 |
| M527T1_17 | 3.5213 | 527.10794 | [M+Cl]- | 492.1387 | N-(4-chlorophenyl)-2-[[5-[(2,6-dimethylphenoxy)methyl]-4-(4-methylphenyl)-1,2,4-triazol-3-yl]sulfanyl]acetamide | C26H25ClN4O2S | 0 |
| M512T1_12 | 3.5127 | 512.099462 | [M+Cl]- | 477.1315 | Flutropium bromide | C24H29BrFNO3 | 2 |
| M195T1_4 | 3.2763 | 194.8902899 | [M-H]- | 195.8997 | Trifluoroiodomethane | CF3I | 10 |
| M281T13_6 | 3.2334 | 281.224263 | [M-H]- | 282.2307 | Diazoacetic acid tetradecyl ester | C16H30N2O2 | 2 |
| M341T13_27 | 3.1872 | 341.3494984 | [M+H]+ | 340.3454 | 1,1,3,3-tetrapentylurea | C21H44N2O | 9 |
| M338T14_15 | 3.1332 | 338.3130258 | [M+Na]+ | 315.3217 | Arachidic acid(d3) | C20H37D3O2 | 6 |
| M658T1_12 | 3.1094 | 658.3349903 | [M+Cl]- | 623.3683 | Apicidin | C34H49N5O6 | 4 |
| M658T1_11 | 3.0796 | 658.3213897 | [M-H]- | 659.3257 | Aripiprazole lauroxil | C36H51Cl2N3O4 | 4 |
| M759T0_8 | 3.0694 | 758.6330519 | [M+Na]+ | 735.6563 | Benzenesulfonate;tetrakis-decylazanium | C46H89NO3S | 16 |
| M467T10_5 | 3.0689 | 467.3040459 | [M+H]+ | 467.3040459 | N-Methyl-N-octadecyl-3-nitro-4-chlorobenzamide | C26H43ClN2O3 | 1 |
| M175T1_11 | 3.0562 | 175.0700452 | [M+H]+ | 174.0632 | 2-chloroethynyl(triethyl)silane | C8H15ClSi | 2 |
| M677T14_14 | 2.9555 | 676.7465509 | [M+Na]+ | 653.7615 | 11-(Pentabromophenoxy)undecan-1-ol | C17H23Br5O2 | 6 |
| M448T1_14 | 2.9534 | 448.3039998 | [M-H]- | 449.3141 | Chenodeoxyglycocholate | C26H43NO5 | 6 |
| M483T10_19 | 2.9159 | 483.4248603 | [M+H]+ | 482.4124 | N-[2-(1H-Indol-1-yl)ethyl]docosanamide | C33H54O2 | 10 |
| M774T15_9 | 2.8322 | 773.6195384 | [M+Na]+ | 750.6315 | Plastoquinol-9 | C53H82O2 | 1 |
| M730T16_7 | 2.8132 | 729.5714123 | [M+H]+ | 728.56 | Trifluoromethanesulfonic acid 2,3-bis(octadecyloxy)propyl ester | C40H79F3O5S | 5 |
| M340T14_36 | 2.8061 | 340.3158195 | [M+H]+ | 339.3137 | Methyl oleoylethanolamide | C21H41NO2 | 15 |
| M445T12_6 | 2.8055 | 445.3820805 | [M+Na]+ | 422.3889 | Phosphine oxide, trioctyl-, dihydrate | C24H55O3P | 8 |

**Supplementary Table S2.** The order of KEGG enriched pathways was displayed based on total compounds and *P*-value.

| Pathways | Total compounds | *P*-value | Enrichment factor |
| --- | --- | --- | --- |
| Amino sugar and nucleotide sugar metabolism* | 20 | 0.006516 | 2.863688 |
| Fructose and mannose metabolism* | 12 | 0.009559 | 3.409013 |
| Polyketide sugar unit biosynthesis* | 2 | 0.014748 | 8.181967 |
| Glycosylphosphatidylinositol (GPI)-anchor biosynthesis* | 12 | 0.047509 | 2.727211 |
| Glycosaminoglycan degradation | 25 | 0.07222 | 1.963608 |
| Glycosaminoglycan biosynthesis - heparan sulfate / heparin | 4 | 0.075003 | 4.0909 |
| Lysine degradation | 14 | 0.079413 | 2.337678 |
| Pentose and glucuronate interconversions | 10 | 0.11171 | 2.45459 |
| Galactose metabolism | 11 | 0.14052 | 2.231479 |
| Steroid biosynthesis | 26 | 0.20134 | 1.573416 |
| Arachidonic acid metabolism | 26 | 0.20134 | 1.573416 |
| Starch and sucrose metabolism | 7 | 0.2062 | 2.33765 |
| Glycosaminoglycan biosynthesis - chondroitin sulfate / dermatan sulfate | 7 | 0.2062 | 2.33765 |
| Pentose phosphate pathway | 27 | 0.22463 | 1.515152 |
| Glycosphingolipid biosynthesis - lacto and neolacto series | 2 | 0.22936 | 4.090983 |
| Glycosphingolipid biosynthesis - globo and isoglobo series | 2 | 0.22936 | 4.090983 |
| Glycerolipid metabolism | 8 | 0.25413 | 2.04545 |
| Porphyrin and chlorophyll metabolism | 23 | 0.30521 | 1.422931 |
| Phosphatidylinositol signaling system | 10 | 0.34995 | 1.636393 |
| Purine metabolism | 65 | 0.3959 | 1.132873 |
| Phosphonate and phosphinate metabolism | 4 | 0.40657 | 2.04545 |
| Glycolysis or Gluconeogenesis | 12 | 0.44149 | 1.363605 |
| Inositol phosphate metabolism | 12 | 0.44149 | 1.363605 |
| alpha-Linolenic acid metabolism | 5 | 0.4794 | 1.636367 |
| Sulfur metabolism | 5 | 0.4794 | 1.636367 |
| Ascorbate and aldarate metabolism | 6 | 0.54339 | 1.363643 |
| Caffeine metabolism | 6 | 0.54339 | 1.363643 |
| Phenylalanine, tyrosine and tryptophan biosynthesis | 6 | 0.54339 | 1.363643 |
| Drug metabolism - cytochrome P450 | 16 | 0.60103 | 1.022704 |
| Terpenoid backbone biosynthesis | 18 | 0.66716 | 0.9090909 |
| Arginine and proline metabolism | 28 | 0.68646 | 0.8766291 |
| Phenylalanine metabolism | 9 | 0.69227 | 0.9090909 |
| Sphingolipid metabolism | 29 | 0.71013 | 0.8464056 |
| N-Glycan biosynthesis | 21 | 0.74966 | 0.7792107 |
| Glycerophospholipid metabolism | 22 | 0.77302 | 0.7437986 |
| Mannose type O-glycan biosynthesis | 12 | 0.79297 | 0.6818027 |
| Drug metabolism - other enzymes | 23 | 0.79448 | 0.7114653 |
| One carbon pool by folate | 13 | 0.81867 | 0.6293662 |
| Aminoacyl-tRNA biosynthesis | 35 | 0.82447 | 0.7012951 |
| Folate biosynthesis | 25 | 0.83216 | 0.6545359 |
| Fatty acid degradation | 14 | 0.84121 | 0.5844194 |
| Nicotinate and nicotinamide metabolism | 14 | 0.84121 | 0.5844194 |
| Pyrimidine metabolism | 47 | 0.85032 | 0.6963303 |
| Glyoxylate and dicarboxylate metabolism | 15 | 0.86097 | 0.5454645 |
| Metabolism of xenobiotics by cytochrome P450 | 17 | 0.89349 | 0.4812783 |
| Alanine, aspartate and glutamate metabolism | 18 | 0.9068 | 0.4545455 |
| Retinol metabolism | 19 | 0.91847 | 0.4306261 |
| Cysteine and methionine metabolism | 33 | 0.92845 | 0.4958719 |
| Tryptophan metabolism | 20 | 0.92869 | 0.4090983 |
| Pantothenate and CoA biosynthesis | 22 | 0.94548 | 0.3718993 |
| Glycine, serine and threonine metabolism | 23 | 0.95235 | 0.3557326 |

**Supplementary Table S3.** Clustered of integrative networks between relative abundance of bacteria and metabolites in the positive and negative modes.

| Community | Bacteria | Node | Code Name (Ion modes) | Input m/z | Database Match |
| --- | --- | --- | --- | --- | --- |
| 1 | *Prevotella, Faecalibacterium, Succinivibrio*, Ruminococcaceae and Lachnospiraceae | Y125 | M299T9_13 (N) | 299.2885 | 2-Octylundecane-1,11-diol |
|  |  | Y149 | M694T10_8 (N) | 694.3559 | Jubanine A |
|  |  | Y156 | M269T1_11 (N) | 268.9833 | Diethyl (2R,3R)-2-bromo-3-fluorobutanedioate |
|  |  | Y162 | M340T10_1 (N) | 340.1761 | Acetylintermedine |
|  |  | Y168 | M674T6_2 (N) | 674.3194 | Jesaconitine |
|  |  | Y170 | M628T10_12 (N) | 628.386 | (4R)-4-Benzyl-3-{(2R,3S,4R,5S,6S)-3-{[tert-butyl(dimethyl)silyl]oxy}-2,4,6-trimethyl-5-[(triethylsilyl)oxy]deca-7,9-dienoyl}-1,3-oxazolidin-2-one |
|  |  | Y21 | M408T12_3 (P) | 408.3089 | Cassaidine |
|  |  | Y59 | M628T10_5 (P) | 628.379 | Pro Glu Arg Val Lys |
|  |  | Y60 | M468T11_39 (P) | 468.3641 | Sulfuric acid, monooctadecyl ester, compd. with 2-(diethylamino)ethanol (1:1) |
|  |  | Y77 | M628T10_6 (P) | 628.3952 | 5-{[3-(Octadecyloxy)propyl]carbamoyl}benzene-1,2,3-triyl triacetate |
| 2 | Enterobacteriaceae | Y131 | M283T15_1 (N) | 283.2637 | 2-hexyldodecanoic acid |
|  |  | Y136 | M596T9_12 (N) | 596.357 | Molybdenum, compd. with nickel (1:8) |
|  |  | Y165 | M526T9_3 (N) | 526.2584 | Glutathionylspermine |
|  |  | Y28 | M291T1_10 (P) | 291.1149 | Butylphosphonic acid diphenyl ester |
|  |  | Y38 | M438T9_10 (P) | 438.337 | (+/-)N-(1-methyl-2-hydroxy-2-phenyl-ethyl) arachidonyl amine |
|  |  | Y4 | M331T12_12 (P) | 331.3266 | Hexadecylmethylglycerol |
|  |  | Y67 | M287T1_6 (P) | 287.0132 | 3,6-Di(ethanesulfonyl)pyridazine |
|  |  | Y75 | M482T10_27 (P) | 482.422 | N-Methyl-N,N-dioctyloctan-1-aminium trifluoroacetate |
|  |  | Y95 | M469T6_2 (N) | 469.0562 | avicularin |
| 3 | *Streptococcus* and *Lactococcus* | Y133 | M387T1_12 (N) | 387.0097 | Ethyl 7-(2,4,5-trichlorophenoxy)heptanoate |
|  |  | Y147 | M221T7_2 (N) | 221.1011 | Thiobenzoic acid S-hexyl ester |
|  |  | Y148 | M640T9_10 (N) | 640.3491 | Leu Leu Val Val Tyr |
|  |  | Y172 | M616T13_5 (N) | 615.5433 | Dioctadecyl thiourea |
|  |  | Y178 | M832T14_4 (N) | 831.5859 | 16:2-Glc-Campesterol |
|  |  | Y182 | M409T10_2 (N) | 409.2349 | LPA(0:0/16:0) |
|  |  | Y36 | M670T10_7 (P) | 670.3949 | L-Phenylalanylglycyl-L-leucyl-L-alanyl-L-lysyl-L-leucine |
|  |  | Y62 | M487T11_9 (P) | 487.2923 | Gln Ile Gln Val |
|  |  | Y124 | M628T10_10 (N) | 628.3551 | Arg Val Ile Trp Gly |
|  |  | Y127 | M803T0_6 (N) | 802.6196 | PE(16:0/24:0) |
|  |  | Y150 | M382T13_10 (N) | 382.2187 | 6,7-Dihydro-8-phenyl-9-(4-(2-(dimethylamino)ethoxy)phenyl)-5-H-benzocycloheptene |
|  |  | Y159 | M441T10_3 (N) | 441.2532 | N-linolenoyl-glutamine |
|  |  | Y174 | M236T0_1 (N) | 235.9143 | Selenophene-2-carbonyl azide |
|  |  | Y177 | M507T10_5 (N) | 507.3006 | Reticulataxanthinz |
|  |  | Y112 | M300T1_6 (N) | 300.0495 | N-Acetyl-D-galactosamine 6-phosphate |
|  |  | Y142 | M630T10_8 (N) | 630.3741 | Jurubine |
|  |  | Y153 | M480T9_3 (N) | 480.2776 | Urea, N-(2,6-bis(1-methylethyl)phenyl)-N'-((1-(1,3-dimethyl-1H-indol-2-yl)cyclopentyl)methyl)- |
|  |  | Y17 | M783T17_4 (P) | 782.5792 | PC(20:3(8Z,11Z,14Z)/16:1(9Z)) |
|  |  | Y18 | M359T13_11 (P) | 359.2989 | Propanoic acid, 3-mercapto-, octadecyl ester |
|  |  | Y181 | M830T0_8 (N) | 829.6348 | 2,3,4,5-Tetraiodo-6-[(naphthalen-1-yl)carbamoyl]benzoic acid |
|  |  | Y100 | M195T1_4 (N) | 194.8903 | Trifluoroiodomethane |
|  |  | Y103 | M658T1_11 (N) | 658.3214 | Aripiprazole lauroxil |
|  |  | Y114 | M658T6_4 (N) | 658.3427 | Arg Arg Arg His |
|  |  | Y12 | M774T15_9 (P) | 773.6195 | Plastoquinol-9 |
|  |  | Y16 | M345T14_12 (P) | 345.2968 | 2-Phenyl-4-tetradecyl-1,3,2-dioxaborolane |
|  |  | Y33 | M339T12_9 (P) | 339.3213 | 13(Z)-Docosenoic Acid |
|  |  | Y8 | M467T10_5 (P) | 467.304 | N-Methyl-N-octadecyl-3-nitro-4-chlorobenzamide |
|  |  | Y93 | M446T1_17 (N) | 446.2883 | Hexyldioxodecyl methyl tyrosinate |
|  |  | Y90 | M512T6_3 (N) | 512.0993 | 1-phenylpyrazole;titanium(3+) |
|  |  | Y91 | M568T6_2 (N) | 568.1222 | 4',4''-Di-2-imidazolin-2-yl-2-nitroterephthalanilide dihydrochloride |
|  |  | Y92 | M535T6_2 (N) | 535.1098 | Haploside A |
|  |  | Y7 | M759T0_8 (P) | 758.6331 | Benzenesulfonate;tetrakis-decylazanium |
|  |  | Y69 | M522T9_21 (P) | 522.3917 | 3-[3,4-Bis(decyloxy)phenyl]-2-(ethoxymethyl)prop-2-enenitrile |
|  |  | Y61 | M487T11_6 (P) | 487.2788 | Pristimerin |
|  |  | Y39 | M265T1_6 (P) | 265.0237 | Azelaic acid, potassium salt |
|  |  | Y27 | M603T11_4 (P) | 603.4063 | (3beta)-3-{[(2Z)-3-(4-Hydroxyphenyl)prop-2-enoyl]oxy}urs-12-en-28-oic acid |
|  |  | Y2 | M440T9_13 (P) | 440.282 | Ethanesulfonic acid, 2-(cyclohexyl(1-oxohexadecyl)amino)-, sodium salt |
|  |  | Y19 | M469T11_35 (P) | 469.3929 | Lupeol acetate |
|  |  | Y140 | M339T10_10 (N) | 339.2299 | 2,4-Bis(4-methylpiperazin-1-yl)-3H-1,5-benzodiazepine |
|  |  | Y130 | M668T10_9 (N) | 668.3775 | 3-(4-Methylpyridin-1-ium-1-yl)cholest-5-ene 4-methylbenzene-1-sulfonate |
|  |  | Y11 | M483T10_19 (P) | 483.4249 | N-[2-(1H-Indol-1-yl)ethyl]docosanamide |
|  |  | Y107 | M568T1_8 (N) | 568.1172 | Idarubicin hydrochloride |
|  |  | Y106 | M527T1_15 (N) | 527.0965 | Malvidin 3-O-glucoside |
|  |  | Y1 | M529T6_2 (P) | 529.1208 | Triphenylstannyl 4-tert-butylbenzoate |
|  |  | Y96 | M512T6_2 (N) | 512.084 | 8-Methylthiooctyl glucosinolate |
|  |  | Y98 | M527T1_17 (N) | 527.1079 | N-(4-chlorophenyl)-2-[[5-[(2,6-dimethylphenoxy)methyl]-4-(4-methylphenyl)-1,2,4-triazol-3-yl]sulfanyl]acetamide |
|  |  | Y99 | M512T1_12 (N) | 512.0995 | Flutropium bromide |
| 4 | *Dialister* | Y155 | M307T13_6 (N) | 307.3084 | Glyodin |
|  |  | Y37 | M631T10_8 (P) | 631.4385 | Dexamethasone palmitate |
|  |  | Y88 | M225T1_9 (P) | 225.0483 | Penmacric acid |
| 5 | *Lactobacillus* | Y14 | M340T14_36 (P) | 340.3158 | (4e)-4-(hexadecylimino)pentanoic acid |
|  |  | Y42 | M630T10_4 (P) | 630.3835 | Trp Leu Arg Arg |
|  |  | Y49 | M319T9_1 (P) | 319.2234 | 3-Oxooctadec-4-enoic acid |
|  |  | Y76 | M338T14_12 (P) | 338.2501 | Suloctidil |
|  |  | Y79 | M602T9_9 (P) | 602.3488 | Asp Arg Arg Arg |
|  |  | Y83 | M598T9_6 (P) | 598.3732 | Lysophosphatidylcholine (22:2) |

**Supplementary Table S4.** Table of the characteristics of gut ascariasis patient (n = 19) in Ban Mae Salid Luang (BL) and Gre Key (GK) village, Thailand.

| Sample code | Age of human hosts | Sex of human hosts | Infection intensity | Collection date | Geographic location |
| --- | --- | --- | --- | --- | --- |
| AL03 | 3 | Male | Moderate | 06/22/2019 | Thailand: BLvillage |
| AL17 | 8 | Male | Moderate | 06/24/2019 | Thailand: BLvillage |
| AL25 | 9 | Male | Heavy | 06/26/2019 | Thailand: BLvillage |
| AL26 | 9 | Male | Heavy | 06/28/2019 | Thailand: BLvillage |
| AL29 | 13 | Female | Moderate | 06/30/2019 | Thailand: BLvillage |
| AL32 | 11 | Male | Heavy | 07/02/2019 | Thailand: BLvillage |
| AL35 | 9 | Male | Moderate | 07/04/2019 | Thailand: BLvillage |
| AL38 | 8 | Male | Moderate | 07/06/2019 | Thailand: BLvillage |
| AL40 | 9 | Male | Moderate | 07/08/2019 | Thailand: BLvillage |
| AL43 | 8 | Male | Moderate | 07/10/2019 | Thailand: BLvillage |
| AL44 | 8 | Male | Moderate | 07/12/2019 | Thailand: BLvillage |
| AL45 | 4 | Female | Light | 07/14/2019 | Thailand: GKvillage |
| AL46 | 4 | Female | Light | 07/16/2019 | Thailand: GKvillage |
| AL48 | 22 | Female | Moderate | 07/18/2019 | Thailand: GKvillage |
| AL50 | 24 | Female | Moderate | 07/20/2019 | Thailand: GKvillage |
| AL51 | 24 | Female | Moderate | 07/22/2019 | Thailand: GKvillage |
| AL52 | 8 | Female | Light | 07/24/2019 | Thailand: GKvillage |
| AL53 | 9 | Female | Heavy | 07/26/2019 | Thailand: GKvillage |
| AL54 | 22 | Female | Moderate | 07/28/2019 | Thailand: GKvillage |
